# The expression of a viral microRNA is regulated by clustering to allow optimal B cell transformation

**DOI:** 10.1093/nar/gkv1330

**Published:** 2015-12-03

**Authors:** Janina Haar, Maud Contrant, Katharina Bernhardt, Regina Feederle, Sven Diederichs, Sébastien Pfeffer, Henri-Jacques Delecluse

**Affiliations:** 1 Pathogenesis of Virus Associated Tumors, German Cancer Research Center, Im Neuenheimer Feld 242, 69120 Heidelberg, Germany; 2 Inserm unit U1074, Im Neuenheimer Feld 242, 69120 Heidelberg, Germany; 3 Architecture et Réactivité de l'ARN – UPR 9002, Institut de Biologie Moléculaire et Cellulaire du CNRS, Université de Strasbourg, 15 rue René Descartes, F-67084 Strasbourg Cedex, France; 4 Division of Cancer Research, Clinic for Thoracic Surgery, University Hospital Freiburg, Breisacher Str. 86b, 79110 Freiburg, Germany; 5 Division of RNA Biology & Cancer, German Cancer Research Center, Im Neuenheimer Feld 280, 69120 Heidelberg, Germany & Institute of Pathology, University Hospital Heidelberg, Im Neuenheimer Feld 224, 69120 Heidelberg, Germany

## Abstract

The Epstein-Barr virus (EBV) transforms B cells by expressing latent proteins and the BHRF1 microRNA cluster. MiR-BHRF1–3, its most transforming member, belongs to the recently identified group of weakly expressed microRNAs. We show here that miR-BHRF1–3 displays an unusually low propensity to form a stem–loop structure, an effect potentiated by miR-BHRF1–3's proximity to the BHRF1 polyA site. Cloning miR-BHRF1–2 or a cellular microRNA, but not a ribozyme, 5′ of miR-BHRF1–3 markedly enhanced its expression. However, a virus carrying mutated miR-BHRF1–2 seed regions expressed miR-BHRF1–3 at normal levels and was fully transforming. Therefore, miR-BHRF1–2's role during transformation is independent of its seed regions, revealing a new microRNA function. Increasing the distance between miR-BHRF1–2 and miR-BHRF1–3 in EBV enhanced miR-BHRF1–3's expression but decreased its transforming potential. Thus, the expression of some microRNAs must be restricted to a narrow range, as achieved by placing miR-BHRF1–3 under the control of miR-BHRF1–2.

## INTRODUCTION

MicroRNAs (miRNAs) are small regulatory RNAs that serve crucial roles in a wide range of cellular processes such as differentiation, immune and inflammatory signaling, proliferation or apoptosis (1–4) and are commonly deregulated in cancer (5,6). MiRNAs are processed from primary transcripts that comprise a double-stranded miRNA stem–loop that is targeted and cleaved in the nucleus by the Microprocessor complex containing the RNase III enzyme Drosha and DiGeorge syndrome critical region 8 (DGCR8) (7,8). The cleavage liberates a pre-miRNA hairpin which is exported by Exportin 5 (9) and further processed by Dicer in the cytoplasm to yield a 19–22nt long mature miRNA that is incorporated into the RNA-induced silencing complex (RISC) with an Argonaute protein (10,11). The nucleotides 2–8 of a mature miRNA are termed the seed region and are crucial for recognition of most target RNAs, while the remaining sequence has only partial complementarity (12). This enables miRNAs to interact with hundreds of potential target genes (13) and makes them ideal tools for manipulation of host cell processes by viruses.

The first viral miRNAs were identified in the Epstein-Barr virus (EBV) (14). EBV is an oncogenic virus that causes multiple types of lymphomas and carcinomas (15–17). EBV can transform resting primary B cells with very high efficiency (18). This process requires co-expression of the viral latent genes as well as of the BHRF1 miRNA cluster that comprises three members (miR-BHRF1–1 to -3). Cells immortalized with a mutant that lacks the miR-BHRF1 cluster grow more slowly, show an abnormal cell cycle distribution and undergo apoptosis more frequently (19,20). Several cross-linking and immunoprecipitation (CLIP) screens have identified potential cellular targets for the BHRF1 miRNAs (21–23), but which of these genes represent the key targets of the BHRF1 miRNAs remains to be determined. The EBV BART miRNA cluster has also been suggested to contribute to transformation (24,25). Interestingly, the EBV miRNAs are mainly required at the beginning of the transformation process and infection of humanized mice with a mutant lacking the BHRF1 miRNAs results in a tumor incidence similar to wt-infected mice, although acute systemic EBV infection is more pronounced in the presence of the BHRF1 cluster (26). In the absence of the BHRF1 miRNAs, the transformation efficiency of the virus drops approximately 20 fold, and this effect can be mainly ascribed to miR-BHRF1–2 and miR-BHRF1–3 (27). However, B cells infected with a mutant virus that lacks miR-BHRF1–2 express markedly reduced levels of miR-BHRF1–3, suggesting that wild type expression of miR-BHRF1–3 requires miR-BHRF1–2.

In the present paper, we show that miR-BHRF1–3 alone is processed with low efficiency and that miR-BHRF1–2's main contribution to the transformation process is to enhance miR-BHRF1–3 expression. Moreover, we investigate the molecular mechanisms that control expression of miR-BHRF1–3 and show that they are important to maintain the transforming properties of the virus.

## MATERIALS AND METHODS

### Cell lines and cell culture

WI38 are human primary embryonic lung fibroblasts (28). Raji is an EBV-positive cell line established from a Burkitt's lymphoma (29). HEK293 cells are derived from human embryonic kidney cells by adenovirus transformation (30,31). All cell lines were kept in RPMI 1640 medium (Gibco) supplemented with 10% fetal bovine serum (FBS, Sigma). Primary human B cells were isolated by positive selection with CD19 Dynabeads (Invitrogen) from buffy coats after density gradient centrifugation of whole blood on a Ficoll cushion. For generation of lymphoblastoid cell lines (LCLs), 5 × 10^5^ B cells were infected at a multiplicity of infection (MOI) of five according to qPCR viral titers and subsequently kept in RPMI 1640 with 10% FBS until outgrowth.

### Construction of miRNA expression plasmids

All plasmids for miRNA analysis were cloned into the mammalian expression vector pcDNA 3.1 (+). For a description of plasmid inserts and cloning methods, please refer to Supplementary Experimental Procedures and Supplementary Table S1.

### Construction of EBV mutants

Recombinant viruses were cloned by chromosomal building or *En passant* mutagenesis of BAC DNA. For cloning details, please refer to Supplementary Experimental Procedures.

### Generation of viral supernatants and viral titer determination

Viral BAC DNA was stably transfected into HEK293 cells using Hygromycin B selection. Production of viruses was triggered by transfection of BZLF1 and BALF4 expression plasmids. Titers of obtained viral supernatants were determined by TaqMan qPCR or infection of Raji cells to determine the green Raji unit functional titer (gru). All steps are described in detail in Supplementary Experimental Procedures.

### B cell transformation assay

To determine the efficacy of B cell transformation upon viral infection, primary B cells were infected at an MOI of 0.01 gru and seeded at a concentration of 10^2^ cells per well in 96-U wells, which were previously coated with 50Gy-irradiated WI38 cells. At 31 days post infection (dpi), wells with visible proliferation of B cells were counted as transformed.

### miRNA expression studies

For BHRF1 miRNA expression analysis, HEK293 cells were seeded at a density of 2 × 10^5^ cells per six-well and transfected the following day with 1 μg plasmid DNA using Metafectene. 0.2 μg pEGFP-C1 was co-transfected per well to normalize single experiments for differences in transfection efficiency. Medium was changed the next day, cells harvested 48 h after transfection, washed once in PBS and resuspended in 1ml PBS. 900 μl of cell suspension were taken for RNA extraction and 100μl subjected to FACS analysis of GFP-positive cells on a FACSCalibur™ flow cytometer. To determine miRNA stability, transfected cells were treated after changing medium with 5μg/ml Actinomycin D (Serva) for indicated times prior to harvesting.

### RNA preparation

Total RNA from LCLs or transfected HEK293 cells was extracted with TRIzol reagent (Ambion) according to the manufacturer's protocol. RNA pellets were dissolved in 50μl H_2_O, concentration and purity determined on a NanoDrop2000 spectrophotometer (Thermo Scientific) and all samples adjusted to 400 ng/μl for subsequent experiments.

*In vitro* RNA (IVR) transcription was performed with the TranscriptAid T7 High Yield Transcription Kit (Thermo Scientific) according to the manufacturer's protocol with corresponding pcDNA3.1 (+)-derived miRNA constructs as a template, which were previously linearized at the 3′ end with EcoRV. pcDNA3.1 (+) contains a T7 promoter. Sequences of *in vitro* transcripts are shown in Supplementary Table S3. IVR samples were purified by Phenol-chloroform extraction and RNA integrity monitored on 8% TBE acrylamide gels. Prior to each experiment, RNA was denatured at 95°C for 3 min, cooled down on ice for 3 min and folded in 1× structure buffer (10 mM Tris–HCl, 80 mM KCl, 10 mM MgCl_2_, pH 7.0, supplemented with 400 u/ml RNAsin (Promega)) at 37°C for 30 min.

### miRNA analysis by stem–loop RT-PCR

Quantification of miRNAs by stem–loop RT-PCR was described previously (32). RT and stem–loop PCR primer sequences for BHRF1 miRNAs were as reported (33). Reverse transcription of all miRNAs was performed in one reaction mix containing 12.5 nM of each RT primer with the TaqMan MicroRNA Reverse Transcription Kit (Applied Biosystems) according to kit instructions. After reverse transcription, the concentration was adjusted to obtain 2 ng/μl template for stem–loop PCR. Each 20 μl PCR contained 10 μl of 2× TaqMan Universal PCR Master Mix (Applied Biosystems), 5 μl of RT product, 1.5 μM forward primer, 0.7 μM universal reverse primer and 0.2 μM probe. TaqMan MicroRNA RNU48 Assay (Applied Biosystems) was used for normalization. Samples were incubated at 95°C for 10 min followed by 40 cycles of 95°C for 15 s and 56°C for 1 min. For each miRNA, measurements were performed in duplicates. Triplicate transfection experiments were analyzed within one PCR plate, normalized to one of three wt values according to ΔΔCt quantification and corrected for differences in the percentage of GFP-positive cells for HEK293 transfections. Unless specified otherwise, figures show average values of three experiments with standard deviation (SD) error bars.

### Microprocessor cleavage assay

To monitor cleavage of BHRF1 miRNA precursors, an *in vitro* cleavage assay was performed. First, HEK293 cells were transfected on a 100 mm cell culture plate with 8 μg Flag-Drosha and 2 μg HA-DGCR8 expression plasmids using Metafectene (Biontex). Medium was changed the following day. Cells were harvested 48 h post-transfection, washed once with 5 ml ice-cold PBS and lysed in 300 μl ice-cold lysis buffer (20 mM Tris–HCl, 100 mM KCl, 200 μM EDTA, 5% glycerol, 500 μM DTT, 250 μM PMSF, pH 8.0). Flag-Drosha and HA-DGCR8 were described previously (34). The Microprocessor lysate was sonicated five times for 30 s intervals (Sonicator UW2070, Bandelin Electronic) and debris centrifugated at max speed for 10 min in a tabletop centrifuge at 4°C. For each cleavage reaction, a mix in a total reaction volume of 30 μl was prepared containing 6.4 mM MgCl_2_, 1 u/μl RNAsin (Promega), 450fmol of a folded IVR transcript encompassing miR-BHRF1–2 or -3 precursors or both (preparation as described above) and 15 μl of Microprocessor lysate. Samples were incubated at 37°C and snap-frozen at indicated time points on dry ice. Controls containing lysate or RNA only were added to each assay. 50fmol of synthetic miR-BHRF1–2* or -3 was added to RNA only controls of corresponding blots to be able to adjust exposure time of corresponding miRNAs to differences in blot hybridization. Processed RNA was purified after addition of 170 μl elution buffer (300mM sodium acetate, 2% SDS) by phenol-chloroform extraction. MiRNA precursors were detected by Northern blotting as described in Supplementary Experimental Procedures. Northern blot probes and IVR sequences are listed in Supplementary Tables S2 and S3.

### SHAPE analysis

We analyzed the RNA structure by ‘Selective 2′-hydroxyl acylation analyzed by primer extension’ (SHAPE) as described previously (35,36). For BHRF1 RNA modification, 2pmol of folded RNA transcripts were mixed with 2 μg of yeast tRNA (Ambion) and incubated with 90 mM *N*-methylisatoic anhydride (NMIA, Invitrogen) in 12 μl total volume for 40 min at 37°C. Control reactions were treated with anhydrous DMSO only. Reactions were stopped by adding 88 μl H_2_O and RNA purified by ethanol precipitation. Pellets were dissolved in 7 μl TE buffer (10 mM Tris–HCl, 1 mM EDTA, pH 7.5).

Reverse transcription (RT) was performed as described previously (37). A set of four primers (Life Technologies) was used for RT reactions. A primer 5′ labeled with VIC was chosen for modified samples or 5′ labeled with PET for DMSO controls. Additionally, two sequencing reactions were performed with unmodified RNA upon addition of ddGTP or ddATP and a 6-FAM or NED 5′ labeled primer. Primer binding sites were identical for all transcripts as shown in Supplementary Table S3, where primer sequence corresponds to 5′- TTATAGGCCTCACTGGCC -3′. All four RT reactions were pooled, cDNA purified by phenol–chloroform extraction and dissolved in 10 μl deionized formamide. Samples were processed by capillary electrophoresis on an ABI 3130xl Genetic Analyzer, capillary size 50 cm. For RNA secondary structure prediction, electropherograms were analyzed as described previously (38). SHAPE reactivity values for each nucleotide were generated using ShapeFinder 1.0 (39) and served as a basis for modeling of secondary structures with RNAstructure 5.6 (40) using default values for the SHAPE slope (2.6 kcal/mol) and intercept (−0.8 kcal/mol). Final structures were formatted with XRNA 1.0.

Analysis software was downloaded from:

http://bioinfo.unc.edu/Downloads/index.html
http://rna.urmc.rochester.edu/rnastructure.html
http://rna.ucsc.deu/rnacenter/xrna/xrna_download.html

## RESULTS

### MiR-BHRF1–2 and miR-BHRF1–3 are sequentially processed from the same transcript

**Figure 1.**
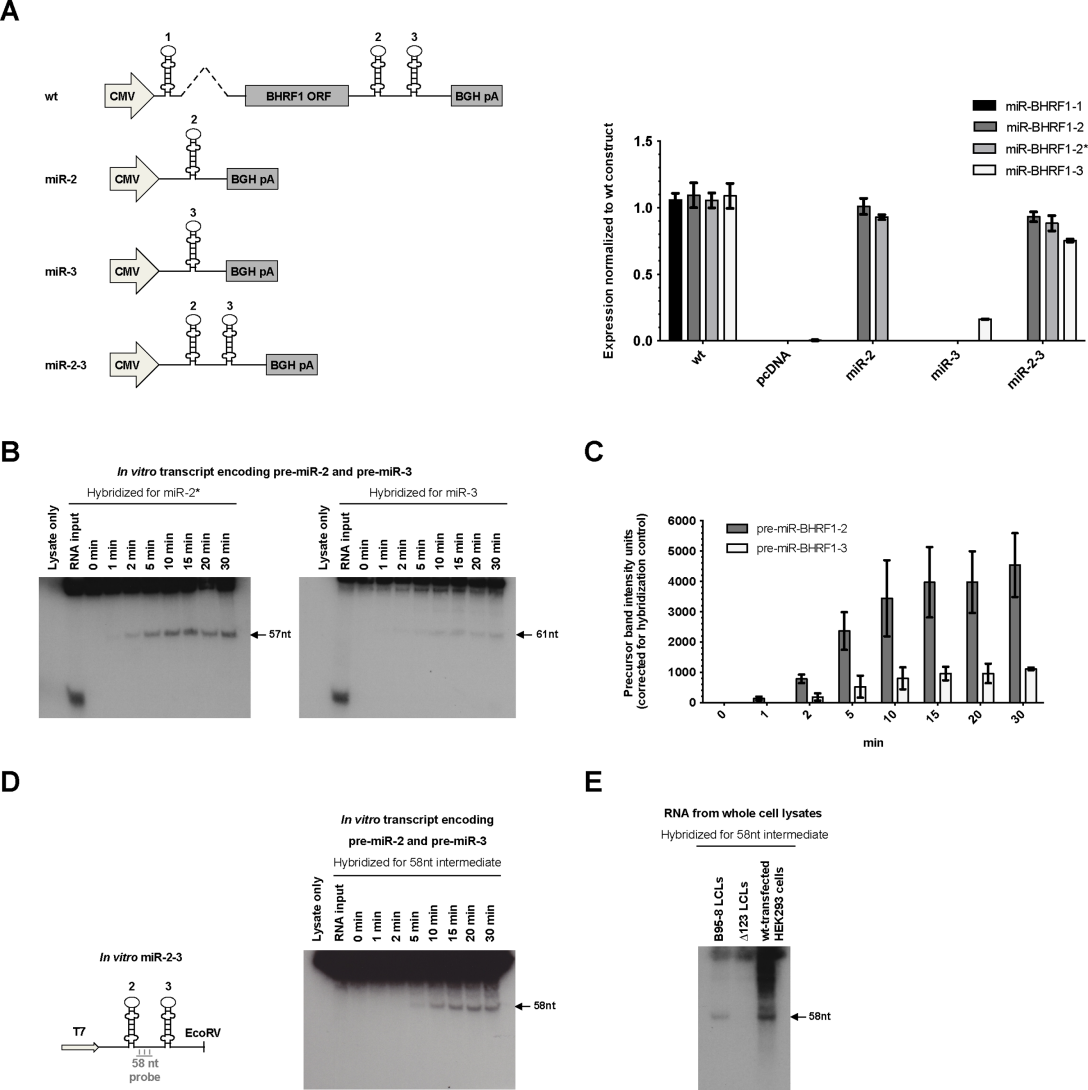
The processing of pre-miR-BHRF1–3 is driven by pre-miR-BHRF1–2. (**A**) Schematics of miRNA expression plasmids used in the study. miRNA expression after transfection of these plasmids in HEK293 cells was quantified by stem–loop RT-PCR (Please see also Supplementary Figure S1). Results show average values from triplicate transfection experiments ± standard deviation (SD). (**B**) Time course of a cleavage assay performed with *in vitro* transcribed RNA carrying pre-miR-BHRF1–2 and -3 showing that pre-miR-BHRF1–2 accumulates more quickly than pre-miR-BHRF1–3 (Please see also Supplementary Figure S2). (**C**) This graph of bars shows the quantification of signals recorded in (B). The mean of the results from three Microprocessor cleavage assays with pre-miR-BHRF1–2 and -3 is shown ± SD. The values are normalized using the signals generated by a spiked synthetic miRNA. (**D**) Hybridization of an *in vitro* cleavage blot with a probe binding to a part of the 58nt long sequence between both miRNAs confirms their processing from a single transcript. (**E**) The 58nt cleavage intermediate can also be detected in lymphoblastoid cell lines (LCLs) and transfected HEK293 cells indicating that endogenous cleavage of both miRNAs also occurs from one transcript.

We previously observed a decrease in miR-BHRF1–3 expression in B cells infected with a virus that lacks pre-miR-BHRF1–2 (27). We set out to determine the molecular mechanisms that underlie the stimulating influence of miR-BHRF1–2 on miR-BHRF1–3 expression by using expression plasmids on which either miR-BHRF1–2 or miR-BHRF1–3 or both are cloned. We precisely quantified miRNA expression using stem–loop RT-PCR and increasing amounts of synthetic miRNAs as standard curves. This absolute quantification confirmed that a plasmid that carries both miR-BHRF1–2 and miR-BHRF1–3 expressed miR-BHRF1–3 at a much higher level than a plasmid that carries miR-BHRF1–3 only (Figure 1A for normalized quantification and Supplementary Figure S1A for absolute values). This assay also revealed that expression plasmids on which the BHRF1 miRNAs are cloned express them at levels that are close to the ones observed in EBV-infected LCLs (Supplementary Figure S1A), although miR-BHRF1–3 levels are closer to those of miR-BHRF1–2 in infected LCLs than they are in HEK293 cells transfected with the expression plasmids. Quantitative miRNA Northern blots confirm these data (Supplementary Figure S1B).

We first assessed the stability of the BHRF1 miRNAs by transfecting HEK293 cells with a plasmid that carries the wild type (wt) BHRF1 locus in the presence of 5 μg/ml Actinomycin D. As expected, levels of BHRF1 transcripts decreased over time. However, this assay did not reveal any differences among any of the BHRF1 miRNAs (Supplementary Figure S1C), as previously observed in LCLs (41). Differences in expression levels of miR-BHRF1–3 can therefore be taken as an indicator of changes in miRNA processing efficiency, based on the assumption that the transcription rate is identical in both plasmids.

The available data suggested that miR-BHRF1–2 is processed before miR-BHRF1–3 in a 5′-3′ direction. We examined this hypothesis in more detail by performing Microprocessor cleavage assays for both miRNA precursors using RNA transcribed *in vitro* from the miR-2–3 expression plasmid as template. We treated the *in vitro* transcribed RNA with whole cell lysates of HEK293 cells transfected with Drosha and DGCR8 expression plasmids and monitored viral pre-miRNA cleavage over time using Northern blotting. We used clearly defined amounts of spiked synthetic miR-BHRF1–2 and -3 to allow precise quantification of the cleaved pre-miRNAs and to allow normalization between experiments. Indeed, the input RNA and high molecular processing intermediates produced a smear upon longer exposure that cannot be reliably quantified (Figure 1 and Supplementary Figure S1A). The analysis of three independent experiments revealed that pre-miR-BHRF1–2 was cleaved earlier and at a higher rate than pre-miR-BHRF1–3 when encoded on the same plasmid (Figure 1B and C). This confirms that a 5′-3′ directional processing of the miRNAs takes place within the cluster (Supplementary Figure S2A). The analysis of pri-miR-BHRF1 processing intermediates generated in these Microprocessor assays added to this evidence; a longer exposure of the Northern blot hybridized with a miR-BHRF1–3 probe revealed the existence of slightly larger RNA fragments that carry unprocessed pre-miR-BHRF1–3 but were not visible on the Northern blot hybridized with the miR-BHRF1–2 probe (Supplementary Figure S2B).

Re-hybridization of the northern blot from Figure 1B with a probe binding to the 58nt sequence located between pre-miR-BHRF1–2 and -3 revealed a band of corresponding size (Figure 1D), thereby confirming cleavage of both precursors from the same transcript under *in vitro* conditions. Furthermore, we could detect this 58nt fragment in LCLs infected with EBV and in HEK293 cells transfected with the wt BHRF1 miRNA expression plasmid, but not in LCLs infected with a virus that lacks the BHRF1 miRNAs (Figure 1E). These data led us to the conclusion that the BHRF1 miRNAs are generated from the same RNA molecule in a 5′ to 3′ manner, with processing of miR-BHRF1–2 preceding miR-BHRF1–3's *in vitro*.

### The miR-BHRF1–3 precursor displays a reduced propensity to form a hairpin

The processing efficiency of a miRNA is strongly correlated to its ability to properly form a hairpin structure (42). Therefore, we used Selective 2′-Hydroxyl Acylation and Primer Extension (SHAPE) (35,38) to precisely determine the structure of the miR-BHRF1–2 and miR-BHRF1–3 stem–loops. Briefly, SHAPE takes advantage of differences in the chemical reactivity of RNA nucleotides when they are paired or unpaired. Unpaired nucleotides can be more easily modified by *N*-methylisatoic anhydride (NMIA) that binds to the free hydroxyl residue on the RNA sugar. The NMIA-nucleotide complex builds a bulky adduct that terminates reverse transcription. Therefore, the secondary structure of a given RNA can be modeled from the obtained extent of nucleotide modification along the transcript.

**Figure 2.**
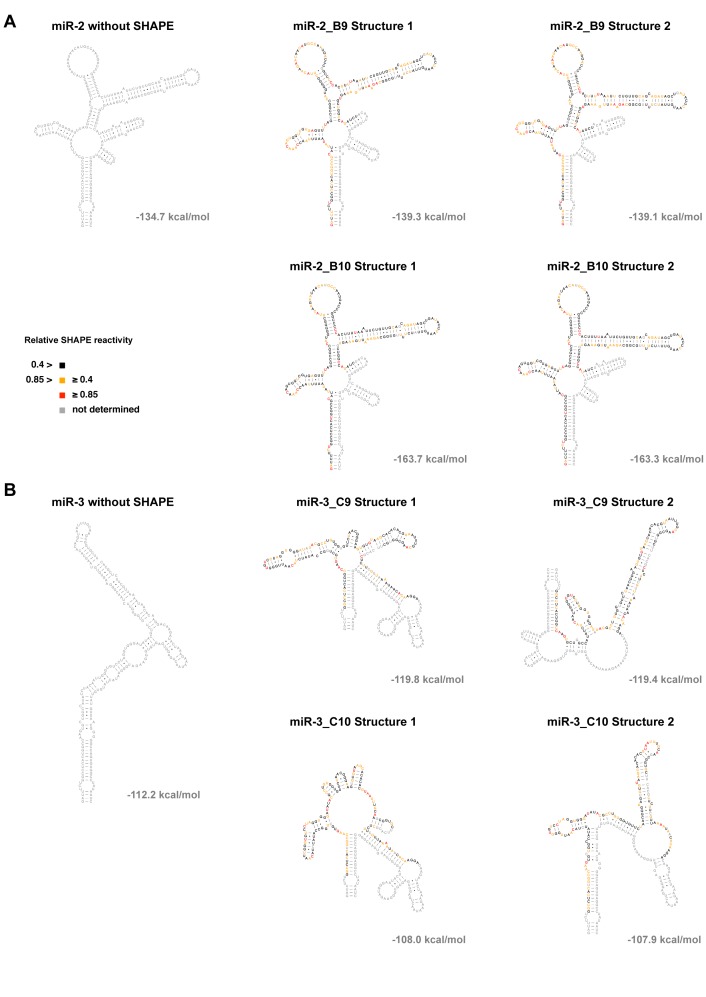
SHAPE analysis of miR-BHRF1–2 and -3 stem–loop structure. The figure shows the two energetically most favorable structures of duplicate SHAPE experiments performed with a transcript encoding (**A**) pri-miR-BHRF1–2 or (**B**) pri-miR-BHRF1–3. The total folding free energy change, ΔG_total_, is provided next to each folded structure.

SHAPE analysis performed on *in vitro* transcribed RNA revealed important differences between both miRNAs. The energetically most favorable structures adopted by miR-BHRF1–2 transcripts were pre-miRNA stem–loops with a double-stranded hairpin (Figure 2A). In contrast, SHAPE data for miR-BHRF1–3 showed that only a sub-fraction of miR-BHRF1–3 transcripts formed a characteristic miRNA stem–loop structure (Figure 2B) that corresponds to 29% and 35% of all predicted miR-BHRF1–3 structures in two independent experiments, in contrast to 92% and 100% for miR-BHRF1–2 replicates (Table 1). The majority of the predicted energetically favorable secondary structures of miR-BHRF1–3 transcripts does not form a pre-miRNA hairpin and is thus highly unlikely to recruit DGCR8 and Drosha efficiently.

**Table 1.** Prevalence of structures containing a microRNA precursor from SHAPE-based analysis of BHRF1 transcripts

Transcript	Number of energetically most favorable structures	Number of structures containing a pre-miRNA	% of ‘processable structures’
miR-2_B9	12	11	92%
miR-2_B10	13	13	100%
miR-3_C9	17	5	29%
miR-3_C10	17	6	35%

SHAPE analysis was performed in duplicates for both miRNAs and percentages were calculated by dividing the number of predicted structures showing a miRNA precursor by the number of all energetically favorable structures.

### MiR-BHRF1–3's intrinsic low expression is partially due to co-transcriptional miRNA processing effects

**Figure 3.**
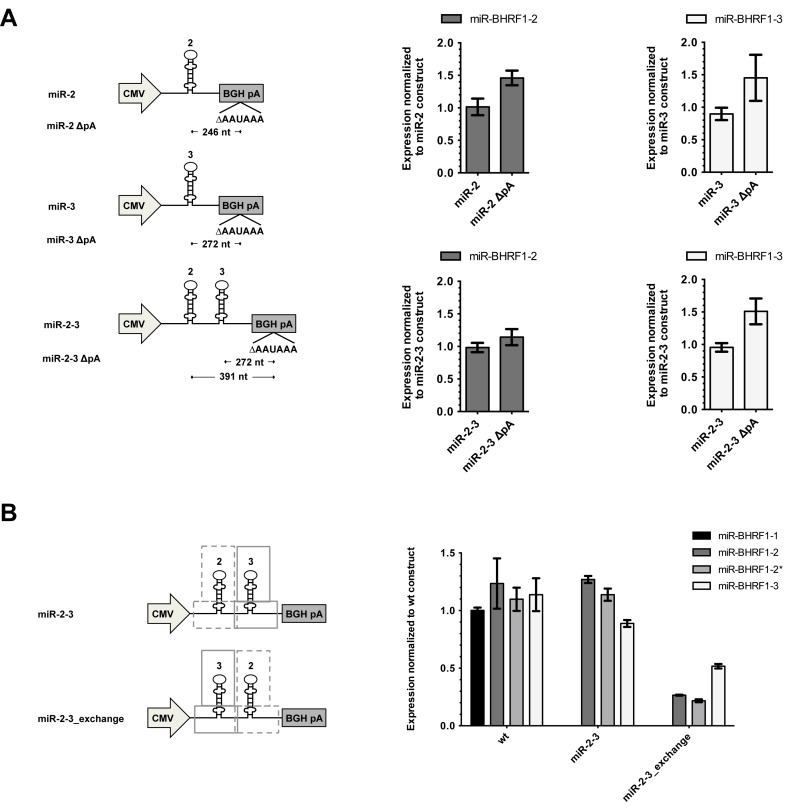
Processing of pre-miR-BHRF1–2 and -3 is linked with transcription. (**A**) To investigate whether Drosha-mediated processing of pre-miR-BHRF1–2 and -3 is coupled to transcription, miRNA expression plasmids with deletion of the polyA cleavage signal (ΔpA) were constructed. miRNA expression changes in transfected HEK293 cells were analyzed by stem–loop RT-PCR. (**B**) Transfection experiments with an expression plasmid containing the same miRNAs but in reciprocal order were performed to confirm that close proximity to the polyA cleavage site is unfavorable as observed by the drop in miR-BHRF1–2 expression. (A and B) Results show average values from triplicate transfections ± SD.

Competition between transcription and pre-miRNA cleavage has previously been reported (43,44). MiRNAs that are located close to the polyA site have less time available for processing than miRNAs located at a more 5′ proximal site within the transcript. Therefore, we examined the role played by the short distance between miR-BHRF1–3 and the transcript polyA cleavage site (272 bp) in HEK293 transfection experiments. A similar short distance is also observed on the viral genome (201 bp). To this end, we compared miR-BHRF1–3 expression from plasmids that carry or do not contain the ‘AAUAAA’ polyA cleavage site 3′ of the BHRF1 miRNAs. Removing the polyA cleavage site lengthens the size of the BHRF1 3′ UTR and could potentially increase processing efficiency. This experiment showed that increasing the distance between miR-BHRF1–3 and the polyA site increased expression by 50% in the presence of miR-BHRF1–2 (Figure 3A). The effect was more limited for miR-BHRF1–2 (15% increase). However, miR-BHRF1–3 is located closer to the BHRF1 polyA site than miR-BHRF1–2. This suggests that processing of BHRF1 miRNAs is coupled to transcription in our experimental setting and is impaired by the short distance between miR-BHRF1–3 and the polyA site, which might not allow timely recognition of a miRNA stem–loop within the nascent transcript.

To further confirm our observations, we exchanged the order of miR-BHRF1–2 and -3 stem–loops including their respective flanking regions. This exchange indeed led to a large decrease in miR-BHRF1–2 expression in transfected HEK293 cells (Figure 3B). Expression of miR-BHRF1–3 also dropped when placed 5′ of miR-BHRF1–2. The most likely explanation is that despite an increased distance to the transcript end, recognition by the Microprocessor complex is inefficient due to the low stem–loop stability of miR-BHRF1–3 observed by SHAPE. MiR-BHRF1–3 processing does not benefit from recruitment of the Microprocessor complex to the site of cleavage by the miR-BHRF1–2 stem–loop, when miR-BHRF1–2 is located 3′, confirming that processing takes place in a 5′ to 3′ direction.

### Sequences surrounding the miRNA stem–loop contribute to miR-BHRF1–3's intrinsic low expression

**Figure 4.**
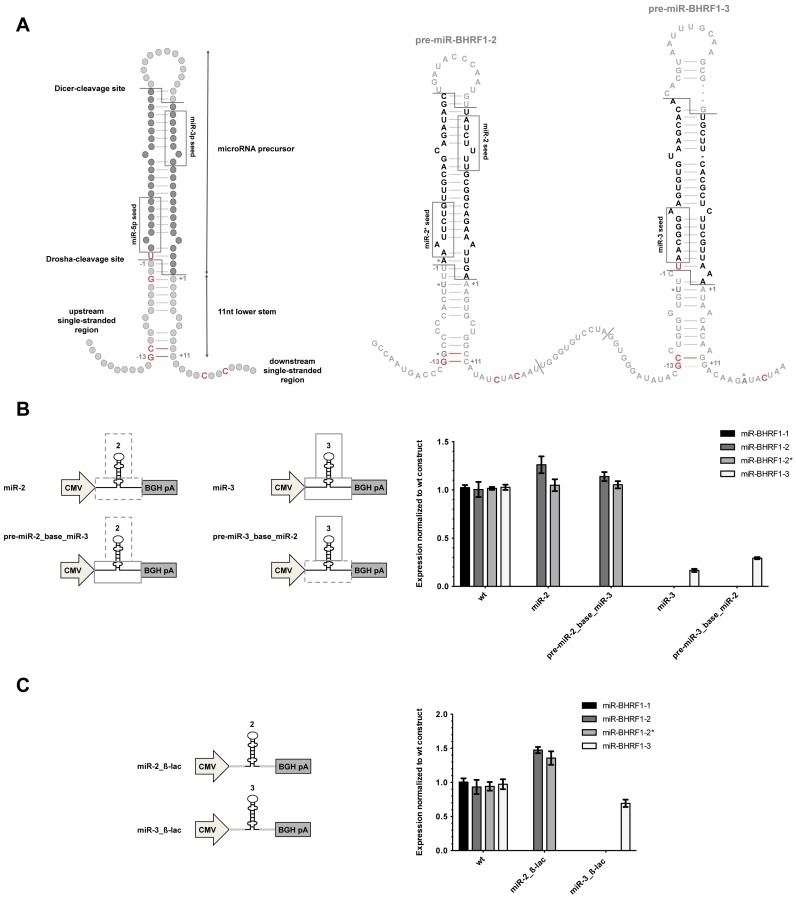
Flanking sequences have an influence on miR-BHRF1–3 expression. (**A**) Schematic overview of the structural and sequence elements so far recognized as necessary for efficient miRNA processing. The location of these nucleotides within the miR-BHRF1–2–3 transcript sequence is highlighted in red; their absence is marked with an asterisk. (**B**) Hybrid miRNAs containing pre-miR-BHRF1–2 and miR-BHRF1–3's base or pre-miR-BHRF1–2 and miR-BHRF1–3's base were constructed. miRNA expression was measured by stem–loop RT-PCR after transfection of the plasmids into HEK293 cells. (**C**) Both miRNA stem–loops were cloned within a fragment of the β-lactamase gene. miRNA expression was determined by stem–loop RT-PCR after transfection of the plasmids into HEK293 cells. (B and C) Results show average values from triplicate transfections ± SD.

We then attempted to link miR-BHRF1–3's structural imperfections with a particular sequence. Previous work has identified structural motifs essential for miRNA processing. This includes the presence of flanking single-stranded sequences 5′ and 3′ of a double-stranded 11nt base region below the pre-miRNA hairpin (45,46). Particular sequence motifs with an impact on pre-miRNA processing have also been recently identified (47,48). They are located underneath the Drosha cleavage site and include a GC at position −13, 5′ of the precursor, with the additional constraint that the nucleotides should be base-paired. Additional motifs include a GNNU starting at position −3, and a CNNC sequence 3′ of the precursor starting between position +16 to +18. An overview of structure and sequence elements needed for efficient miRNA processing and their presence or absence in BHRF1 pri-miRNAs is shown in Figure 4A.

We could observe a paired G at position −13 and the presence of a CUAC sequence 3′ of pre-miR-BHRF1–2. Although a 5′ GC was present before pre-miR-BHRF1–3 as well, the G was unpaired and no CNNC motif was detected. We therefore exchanged the base of miR-BHRF1–2 and miR-BHRF1–3 to analyze the influence of these motifs on BHRF1 miRNAs. This led to a two-fold improvement in miR-BHRF1–3 expression, while miR-BHRF1–2 levels remained unchanged (Figure 4B). The absence of specific sequence elements might therefore contribute to the observed low miR-BHRF1–3 expression, although the effect is moderate.

We also confirmed that the reasons for inefficient expression of miR-BHRF1–3 are unlikely to be found within the stem–loop structure itself by mutating different structure elements inside the precursor. We investigated a potential role played by the terminal loop of miR-BHRF1–3, since there is increasing evidence for processing regulation through binding of regulatory proteins to terminal loop sequences (49), and found that a mutant of miR-BHRF1–3 that carries the miR-BHRF1–2 terminal loop is processed slightly better than its unmodified version, but without reaching wt levels (Supplementary Figure S3A). We also tried to improve the formation of the hairpin by introducing mutations that remove bulges or reinforcing base pairing but this did not improve processing (Supplementary Figure S3B and S3C).

In a further step, we analyzed the influence of more distant surrounding sequences on pre-miR-BHRF1–3 processing. To this end, we cloned the miR-BHRF1–2 and miR-BHRF1–3 stem–loops flanked by unrelated prokaryotic sequences. This increased the expression of both miRNAs, suggesting that the sequences around the BHRF1 gene are generally not favorable for miRNA processing but nevertheless does not explain miR-BHRF1–3's intrinsic weaker production (Figure 4C).

### The presence of an upstream miRNA is required for efficient processing of miR-BHRF1–3 and efficient B cell transformation

**Figure 5.**
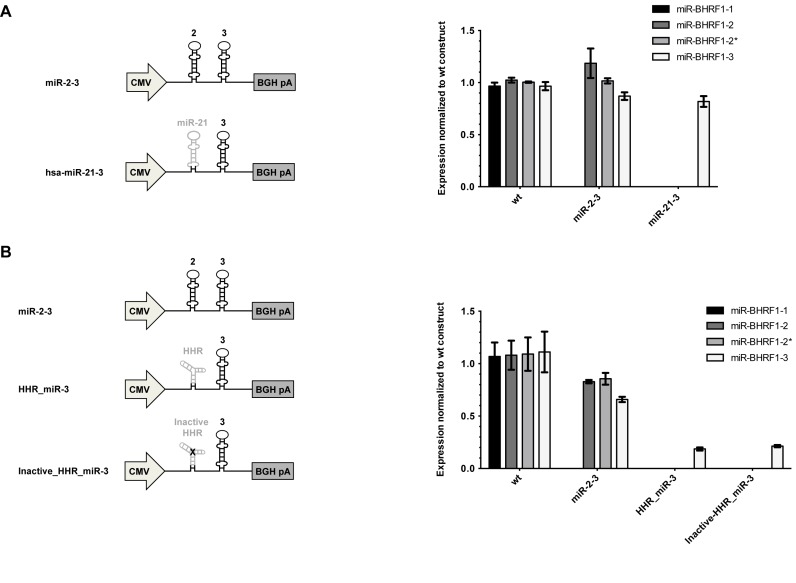
A cellular miRNA can drive normal miR-BHRF1–3 expression. (**A**) Constructs containing pre-miR-BHRF1–2 and -3 or pre-hsa-miR-21 and pre-miR-BHRF1–3 were transfected into HEK293 cells and BHRF1 miRNA levels were measured by stem–loop RT-PCR. (**B**) Similar experiments were conducted with an auto-catalytically cleaving Hammerhead ribozyme (HHR) or a cleavage-deficient counterpart, which was inactivated through a point mutation, placed 5′ of pre-miR-BHRF1–3. The results of the miRNA stem–loop RT-PCR after transfection of the plasmids in HEK293 are indicated (For confirmation of HHR cleavage, please see Supplementary Figure S4). (A and B) Results show average values from triplicate transfections ± SD.

Despite its structural flaws, miR-BHRF1–3 can reach good expression levels, when it is encoded 3′ of miR-BHRF1–2. We wondered whether another miRNA could reproduce this effect and exchanged the miR-BHRF1–2 precursor with pre-hsa-miR-21 and monitored miRNA expression in HEK293 cells. This led to miR-BHRF1–3 expression at levels that did not differ from those observed with miR-BHRF1–2 (Figure 5A). Thus, any efficiently processed miRNA can assume the role played by miR-BHRF1–2 in miR-BHRF1–3 expression. Expression was confirmed with miRNA Northern blots for miR-BHRF1–3 and hsa-miR-21 (Supplementary Figure S4A).

MiRNA processing leads to RNA breaks and we wondered whether this is what stimulates miR-BHRF1–3 expression. Therefore, we cloned a self-cleaving Hammerhead Ribozyme (HHR) (50) 5′ of miR-BHRF1–3. We could confirm that this led to efficient autocatalytic RNA cleavage at the expected HHR site (Supplementary Figure S4B). However, miR-BHRF1–3 located on the cleaved BHRF1 RNA was not processed more efficiently than an intact counterpart containing a cleavage-inactive ribozyme in transfected HEK293 cells (Figure 5B). We therefore conclude that the positive effect on miR-BHRF1–3 expression results from efficient processing of an upstream miRNA and is probably due to Microprocessor recruitment to the site of processing. As a corollary, we concluded that this effect is probably independent of the functionality of the upstream miRNA.

**Figure 6.**
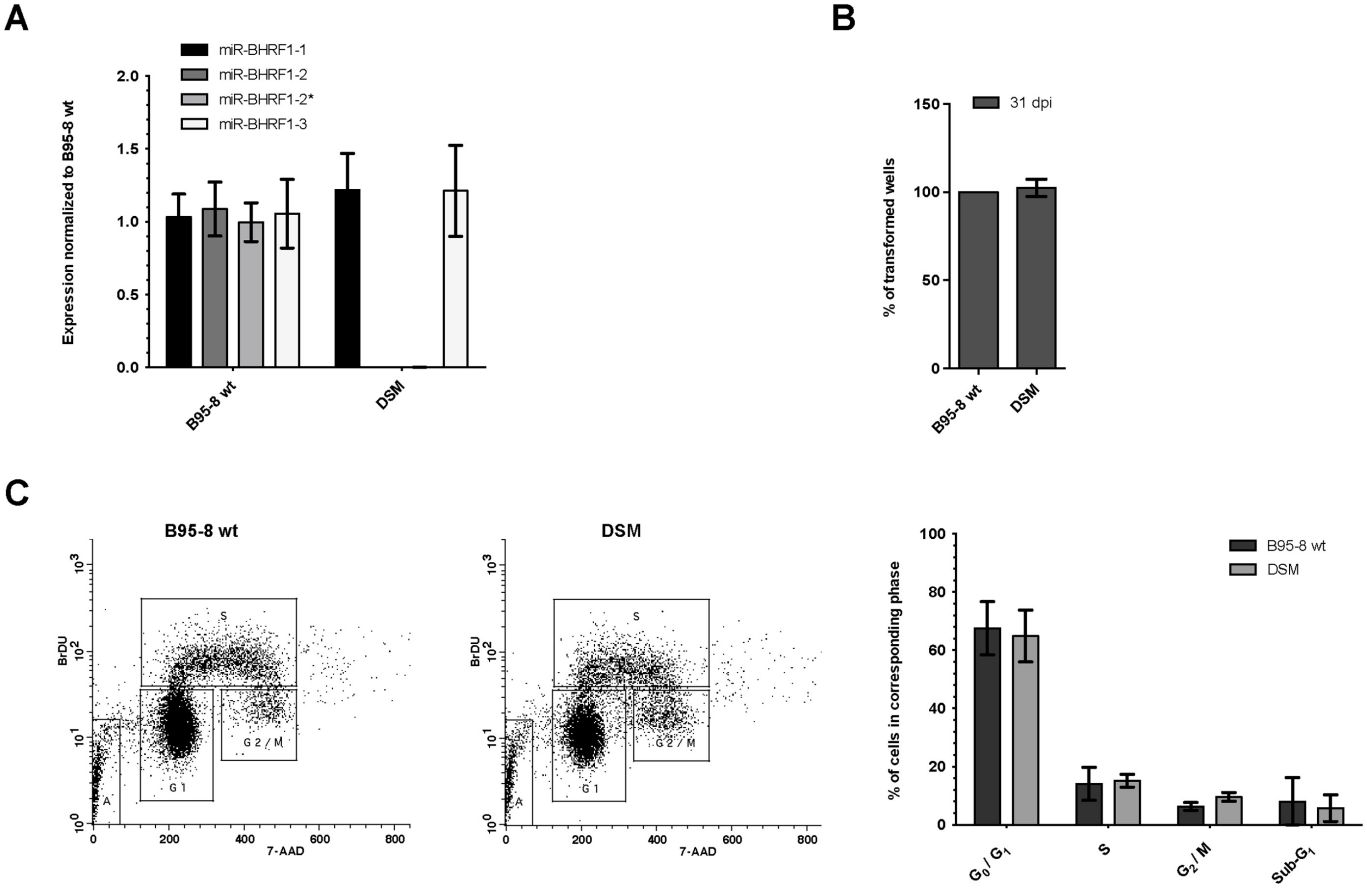
MiR-BHRF1–2 and -2* have no direct impact on B cell transformation by EBV but regulate miR-BHRF1–3 levels. (**A**) miR-BHRF1 expression was analyzed by stem–loop RT-PCR in LCLs generated with B95–8 or double seed mutant (DSM) EBV (please see also Supplementary Figure S5A for the construction of the virus). (**B**) Transformation assays were performed by infection of primary B cells with 0.01 green Raji units (gru) of B95–8 or DSM virus. Outgrowth of infected cells in 96-well plates was monitored and documented at 31 days post infection (dpi). (A and B) LCLs from four different donors were analysed. Average values ± SD are shown. (**C**) A BrdU incorporation assay of B95–8 or DSM LCLs is shown with an example of the measurement (left) and quantification from three experiments ± SD (right).

To confirm this hypothesis, we constructed a virus in which both seed regions of miR-BHRF1–2 and miR-BHRF1–2* were mutated to generate a double seed mutant (miR2/2*DSM). The construction of this virus is detailed in Supplementary Figure S5A. The mutant viral DNA was stably transfected into HEK293 cells to generate HEK293/DSM producer cell lines from which infectious viral stocks could be produced efficiently. LCLs generated with miR2/2*DSM expressed miR-BHRF1–1 and miR-BHRF1–3 at normal levels (Figure 6A). The DSM virus and a wt B95–8 control were used to perform transformation assays at low B cell density within a 96-well cluster plate. No difference between wt and mutant was observed in this assay at 31 days post infection (Figure 6B). Similarly, a BrdU incorporation assay did not reveal any difference in terms of distribution within the cell cycle (Figure 6C). We conclude that miR-BHRF1–2 or -2* do not influence B cell transformation by target-seed interactions. Instead, its main function in B cell transformation seems to be the regulation of miR-BHRF1–3 expression. This represents a hitherto not described function for a microRNA that goes beyond the seed-mediated mRNA targeting.

### Disruption of the sequence between miR-BHRF1–2 and -3 further increases miR-BHRF1–3 processing

**Figure 7.**
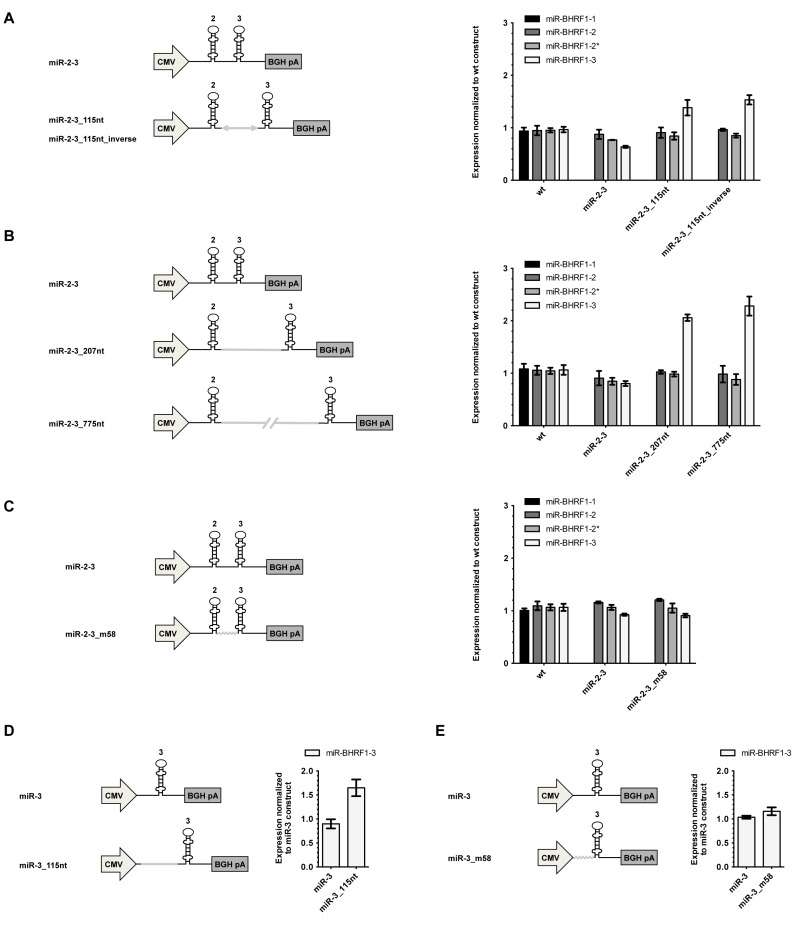
Introduction of spacer sequences 5′ of miR-BHRF1–3 increases its expression. (**A**) A sequence of 115nt was cloned in both orientations between miR-BHRF1–2 and -3 onto an expression plasmid. The constructs were transfected into HEK293 cells to measure BHRF1 miRNA expression by stem–loop RT-PCR. (**B**) The insertion of longer spacers measuring 207nt and 775nt in length between these miRNAs further increased miR-BHRF1–3 expression. (**C**) Partial shuffling of the 58nt intermediate sequence between pre-miR-BHRF1–2 and -3 does not influence miR-BHRF1–3 levels. (**D** and **E**) Similar experiments were performed by changing the sequence 5′ of miR-BHRF1–3 in the absence of miR-BHRF1–2. (A and E) Results show average values from triplicate transfections ± SD.

The distance between pre-miR-BHRF1–2 and pre-miR-BHRF1–3 is only 58nt. This could impose steric constraints on expression of the miRNA located on the 3′ site of the pair. As observed in SHAPE analysis and transfection experiments, sequences surrounding pre-miR-BHRF1–3 negatively influence processing of the miRNA through alternative folding of the transcript. We tested this hypothesis by constructing plasmids in which the distance between both miRNAs is increased through insertion of an 115nt spacer sequence. Transfection assays showed that miR-BHRF1–3 expression increases 1.5-fold with increased distance between both miRNAs (Figure 7A). A similar effect was visible with a plasmid that carries the same spacer, but in the inverse orientation, and increasing the length of spacers led to even higher miR-BHRF1–3 expression (Figure 7B).

Transfections with a plasmid, where a 115nt spacer sequence was inserted in front of miR-BHRF1–3 alone, also result in an increase in miRNA levels, suggesting that disruption of the 5' flanking 58nt sequence contributes to an improved miR-BHRF1–3 processing (Figure 7D). However, a plasmid in which the 58nt between miR-BHRF1–2 and -3 stem–loops is extensively mutated does not influence miR-BHRF1–3 levels neither alone nor in presence of miR-BHRF1–2 (Figure 7C and E).

### Viruses with enhanced miR-BHRF1–3 expression become less transforming

**Figure 8.**
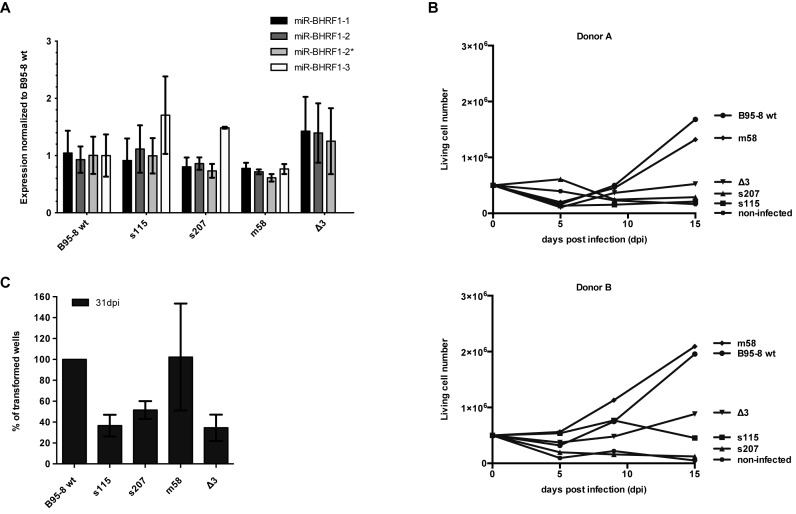
Introduction of spacer sequences 5′ of miR-BHRF1–3 has a negative impact on EBV transforming abilities. (**A**) Expression of BHRF1 miRNAs was measured by stem–loop RT-PCR in LCLs transformed with wild type or a set of mutant B95–8 recombinant viruses. These include two viruses carrying 115nt (s115) and 207nt (s207) spacer inserts between miR-BHRF1–2 and miR-BHRF1–3, as well as a miR-BHRF1–3 knock-out mutant (Δ3) and a virus that carries a mutated 58nt sequence between miR-BHRF1–2 and miR-BHRF1–3 (m58) (please see also Supplementary Figure S5B and S5C for construction of the viruses). LCLs from three different blood samples were included in the analysis whose results are given as average values ± SD. (**B**) Shown are growth curves of B cells from two blood samples transformed by the same set of viruses at high cell density. (**C**) The same experiment was repeated at low cell density and low multiplicity of infection in 96-well cluster plates. The average of the percentages of outgrown wells at 31 days post infection (dpi) ± SD is indicated for four blood samples.

The results gathered so far suggest that miR-BHRF1–3 expression is boosted by the presence of miR-BHRF1–2, but that the close proximity of both miRNAs and the sequence between them actually limits this potentiating effect. This suggests that miR-BHRF1–3's expression needs to be carefully monitored. To test this hypothesis we constructed two viruses in which the distance between both miRNAs was increased by inserting spacer sequences that were either 115nt or 207nt in length (EBV s115, EBV s207) (Supplementary Figure S5B). The assumption was that this should lead to an increase in miR-BHRF1–3 expression and that we would be able to test its consequences on B cell transformation. Furthermore, we constructed a virus in which the 58nt region between the two miRNAs was mutated (EBV m58). Because the external regions of the 58nt sequence are involved in the processing of BHRF1–2 and -3, the mutagenesis was limited to the 24 nucleotides located at the centre of this sequence (Supplementary Figure S5C). Primary B cells were exposed to a set of viruses including both EBV s115 and s207 spacer mutants, EBV m58, EBV Δ3 that lacks miR-BHRF1–3, and a wild type B95–8 virus. Levels of BHRF1 miRNAs were assessed in LCLs obtained from three independent blood samples (Figure 8A). We found that miR-BHRF1–3 was not expressed in cells infected with EBV Δ3 as expected. In contrast, cells infected with the spacer viruses expressed higher levels of miR-BHRF1–3 than cells exposed to wild type viruses. However, spacer insertion did not reduce expression of the anti-apoptotic BHRF1 protein in LCLs generated by infection with s115 and s207, relative to wt levels (Supplementary Figure S6). Cells infected with EBV m58 displayed levels of miR-BHRF1–3 that were only slightly lower than those observed in cells infected with the wild type controls.

We first monitored cell growth at high cell density until 15 dpi and we found that infected B cells grew out more slowly after infection with either of the spacer viruses, relative to wild type controls (Figure 8B). As previously observed, B cells infected with the miR-BHRF1–3 null mutant also grew more slowly than the controls. In contrast, the same B cells exposed to the m58 mutant displayed growth rates that were indistinguishable from those of the B cells infected with the wild type virus.

These results were largely confirmed by transformation assays of B cells at a low cell density and low MOI in 96-well cluster plates. We found that transformation with the spacer viruses was approximately three times less efficient than with the wt virus (Figure 8C) and that these viruses achieved transformation rates that are close to those observed with the miR-BHRF1–3 deletion mutant (27). However, this effect cannot be ascribed to a reduced expression of the BHRF1 protein in the infected cells (Supplementary Figure S6). We also find that the EBV m58 virus is globally endowed with the same transformation abilities as the wild type virus, although the transformation rates varied between donors more markedly that with the other mutants. Taken together with the results of experiments at high cell density, these results indicate that the 58nt region has no substantial influence on the transformation process. We also conclude that a tight regulation of miR-BHRF1–3 expression is crucial for an efficient EBV-mediated B cell transformation and that this fine-tuning is achieved through a well-balanced coupling of miR-BHRF1–2 and -3 processing within the BHRF1 miRNA cluster.

## DISCUSSION

Most herpesviruses encode multiple miRNAs and these have been shown to play key roles in viral infection such as the control of virus lytic replication (51,52), apoptosis of host cells (19,24,53) and the regulation of the immune response against the virus (54). MiRNAs regulate EBV's defining feature, the ability of this virus to transform resting B cells (19,20,24).

In the present paper, we focused on miR-BHRF1–3 and its regulation. The expression of this miRNA is six times higher within the cluster than when expressed on its own. We identified two main molecular mechanisms responsible for its intrinsically low expression level. First, the proximity of miR-BHRF1–3 to the polyA site of the BHRF1 transcript reduced its expression. This co-transcriptional effect results from a competition between transcription termination and the Microprocessor machinery and has previously been described for the cellular miRNAs let-7, lin-4 and hsa-miR-26 (43). Coupling of pre-miRNA processing to transcription has also been shown for miRNAs located in introns of protein-coding genes or within other transcripts (43,44). Second, the miR-BHRF1–3 sequence itself also accounts for its inefficient processing. Our data indicate that motifs in the single-stranded RNA regions before and after the stem–loop and in the terminal loop could play some moderate role in this process. More importantly, the SHAPE analysis (38) has clearly shown that a part of miR-BHRF1–3 together with the RNA region located in the 58nt sequence directly 5′ of miR-BHRF1–3 build double-stranded regions that are thermodynamically more favorable than the miRNA stem–loop. Intriguingly, according to a publication by Altuvia *et al*. (55), 7 out of 31 human small cluster regions containing two or more miRNA precursors are also separated by small 57–69nt intermediate sequences. This corresponds to one fifth (22.6%) of all clusters described within the study and indicates that the short distance between miR-BHRF1–2 and miR-BHRF1–3 is not unique (55). Human microRNA clusters with such a build-up include clusters of oncogenic miRNAs such as miR-15b-16–2 (59nt) and its paralogue miR-16–1–15a (57nt) cluster or the miR-17∼92 cluster (35nt to 92nt). Whether the mechanism of regulation we have identified in the miR-BHRF1 cluster extends to cellular miRNAs remains to be investigated. More generally, there is an increasing recognition that cellular miRNAs are also processed with variable efficiency. This has been partially ascribed to the presence of particular domains within the sequence that immediately precedes the miRNA stem–loop (47,48), but our data indicate that the global structure of the pre-miRNA and its surrounding is likely to play a more important role in this process.

The weak cleavage efficiency of miR-BHRF1–3 can be corrected by preceding it with a cellular or a viral miRNA but not by a ribozyme. This indicates that the potentiating effect of miR-BHRF1–2 or hsa-miR-21 does not rest on a mere cleavage of the pri-miRNA directly upstream of miR-BHRF1–3, but rather requires another function mediated by the miRNAs. The fact that miR-BHRF1–2 is processed before miR-BHRF1–3 suggests that the first miRNA helps recruiting the Microprocessor complex to the pri-miRNA that can then sequentially process miR-BHRF1–2 and miR-BHRF1–3. In this context, it is interesting to see that miR-BHRF1–2 does not seem to play an important role through its seed regions, as a virus that carries a miR-BHRF1–2 double seed mutant retains the wild type virus transforming capacity. This does not mean that miR-BHRF1–2 mRNA targets do not exist; they have been identified by CLIP techniques, but simply that they do not influence EBV-mediated transformation *in vitro*, one important but by far not unique mode of virus-cell interaction. Thus, we have shown that miRNAs can play a role that is independent of their seed regions.

We have propounded a model in which miR-BHRF1–2 acts as a non-transformation-specific miRNA that allows processing of a crucial mediator of transformation. Examination of miRNAs in EBV relatives in monkeys supports this model. EBV belongs to the genus of Lymphocryptoviridae within the gammaherpesvirus subfamily, whose members infect a very large number of animals, including primates and many other mammals. A large number of these viruses have been sequenced and the evolution of viral genes can be traced by sequence comparison. The rhesus Lymphocryptovirus (rLCV) infects rhesus macaques and the Cercopithecine herpesvirus 12 (CeHV12) primarily infects Old World baboons. These viruses encode homologs of the BHRF1 miRNAs that have been recently described (23,49). Interestingly, the homology for pre-miR-BHRF1–2 between EBV, rLCV and CeHV12 is generally much higher than for pre-miR-BHRF1–3, including the seed region (7/7 identical bases in the miR-BHRF1–2 seed region of the 3 viruses, and 7/7 or 6/7 for miR-BHRF1–2* versus 3/7 for miR-BHRF1–3). Thus, we can anticipate that the homologs of miR-BHRF1–2 will be efficiently processed and could similarly facilitate processing of their downstream miRNA. One speculative scenario is that functional miR-BHRF1–2 homologs, in that case miRNAs that are efficiently processed, allowed the creation of polymorphic seed regions across different organisms in miR-BHRF1–3, without having to keep a structure that allows efficient processing, as this is overtaken by the preceding miRNA thereby allowing an efficient adjustment of target recognition in different hosts. The length of the sequence between miR-BHRF1–2 and miR-BHRF1–3 is nearly identical in EBV and in these monkey viruses, suggesting that this is an important feature of these miRNAs that was conserved in both human and primate viruses. The high susceptibility of miR-BHRF1–3 expression to experimental mutagenesis of its sequence might reflect the difficulty to achieve fine-tuned expression in a range that is optimal for manipulation of pathways involved in human B cell transformation.

Other modes of regulation of miRNA expression within clusters have been described. For example, the oncogenic miR-17∼92 cluster builds a large and complex structure that brings some of the miRNAs in a peripheral position, which facilitates their processing. In contrast, those miRNAs that are located inside the complex are more weakly expressed, presumably because the Microprocessor machinery cannot efficiently access them (56,57). Furthermore, binding of the hnRNP 1A cofactor selectively boosts the expression of hsa-miR-18a within the cluster (58). Substantial differences in expression levels have also been reported within the Kaposi's Sarcoma-associated herpesvirus (KSHV) K12 miRNA cluster. This was ascribed to differences in the structure and stability of the stem–loops that could be modulated by modifying the sequence of the stem–loop (37).

Why is miR-BHRF1–3 so tightly regulated? The advantage of viral miRNAs is that they can be easily modified and tested in the context of a complete organism. By constructing viruses that contain a spacer between the BHRF1 miRNAs, we obtained miR-BHRF1–3 expression levels above those recorded in cells infected with wild-type viruses (Figure 8A). Transformation experiments showed that an enhanced miR-BHRF1–3 expression decreased the efficiency of transformation, both in bulk B cell infections and in infections at limiting dilution. This suggests that too strong a deregulation of miR-BHRF1–3′s targets impedes cell growth. Combined to our previous observation that deletion or strong reduction in miR-BHRF1–3 expression also hampers transformation, this work provides an example of a viral microRNA whose expression must be tightly controlled to optimally serve its functions. Interestingly, some of the targets that have been proposed for miR-BHRF1–3 include regulators of cell growth (21). There are indications that a miRNA can regulate different subsets of target genes depending on the miRNA concentration within a cell (59). In the same study, it was shown that repression of a given target mRNA does not linearly correlate with miRNA levels, but has an optimum within a narrow range.

Taken together, our study provides an example of a viral miRNA whose expression must be kept within a specified range to optimally serve its functions. This is obtained by including a weakly processed miRNA inside a cluster in close proximity to a well-processed miRNA. The observations described in this study will not only improve our understanding of how viral miRNA expression is controlled but might apply more generally to human miRNA clusters.

## Supplementary Material

SUPPLEMENTARY DATA
