# Supplementary material for: The expression of a viral microRNA is regulated by clustering to allow optimal B cell transformation
Source: Nucleic Acids Res. 2015 Dec 3;44(3):1326–41. doi: 10.1093/nar/gkv1330 (PMC4756819; doi:10.1093/nar/gkv1330)
Supplement: SUPPLEMENTARY DATA [file supp_44_3_1326__index.html]

The expression of a viral microRNA is regulated by clustering to allow optimal B cell transformation — The expression of a viral microRNA is regulated by clustering to allow optimal B cell transformation — SUPPLEMENTARY DATA 

# The expression of a viral microRNA is regulated by clustering to allow optimal B cell transformation

## SUPPLEMENTARY DATA

- SUPPLEMENTARY DATA
