# Supplementary material for: The expression of a viral microRNA is regulated by clustering to allow optimal B cell transformation
Source: Nucleic Acids Res. 2015 Dec 3;44(3):1326–41. doi: 10.1093/nar/gkv1330 (PMC4756819; doi:10.1093/nar/gkv1330)
Supplement: SUPPLEMENTARY DATA [file supp_gkv1330_nar-03072-y-2015-File010.pdf]

The expression of a viral microRNA is regulated by clustering to allow optimal B cell transformation

Janina Haar, Maud Contrant, Katharina Bernhardt, Regina Feederle, Sven Diederichs, Sébastien Pfeffer and Henri-Jacques Delecluse

SUPPLEMENTARY DATA

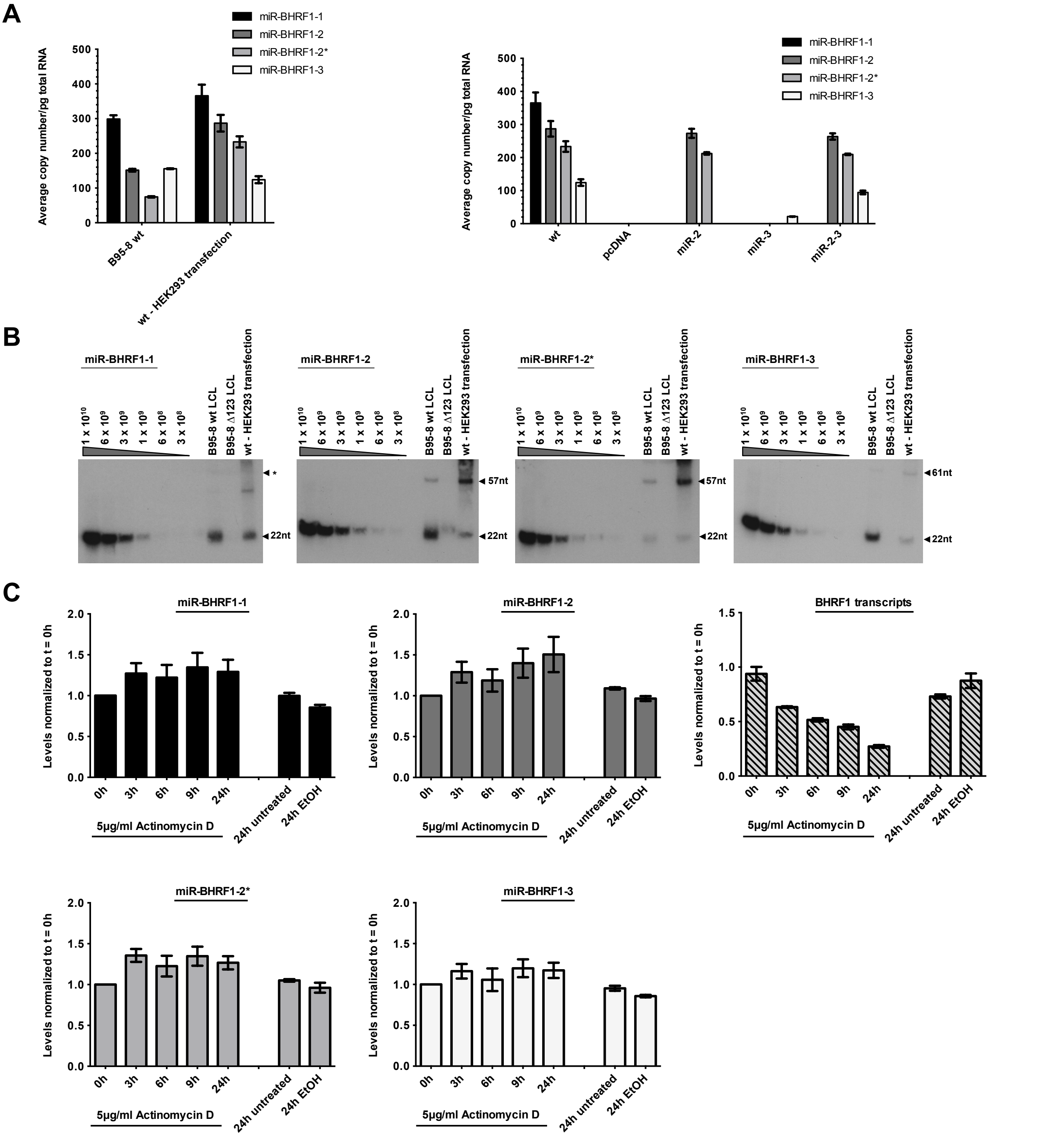

Figure S1. Absolute quantification of BHRF1 microRNA expression in HEK293 cells and B95-8 wt LCLs. **(A)** We assessed absolute expression levels of all BHRF1 miRNAs in HEK293 cells transfected with the plasmids described in Figure 1A and in LCLs generated with B95-8 EBV. Average copy numbers/pg total RNA were calculated from transfection experiments for each miRNA using standard curves generated with synthetic miRNAs. Results show average values from triplicate transfections ± s.d. **(B)** Quantitative microRNA Northern blots with synthetic miRNA standards were performed in parallel to quantify BHRF1 miRNAs in the same cell lines as in (A). * No pre-miR-BHRF1-1 was detected at the expected size of 61nt. **(C)** Analysis of BHRF1 miRNA levels after treatment of HEK293 cells transfected with the wt construct (Figure 1A) with 5µg/ml Actinomycin D. We also recorded the decline of BHRF1 transcript numbers over time to demonstrate the efficacy of transcription inhibition. Results show mean values from duplicate transfection experiments with deviation from mean.

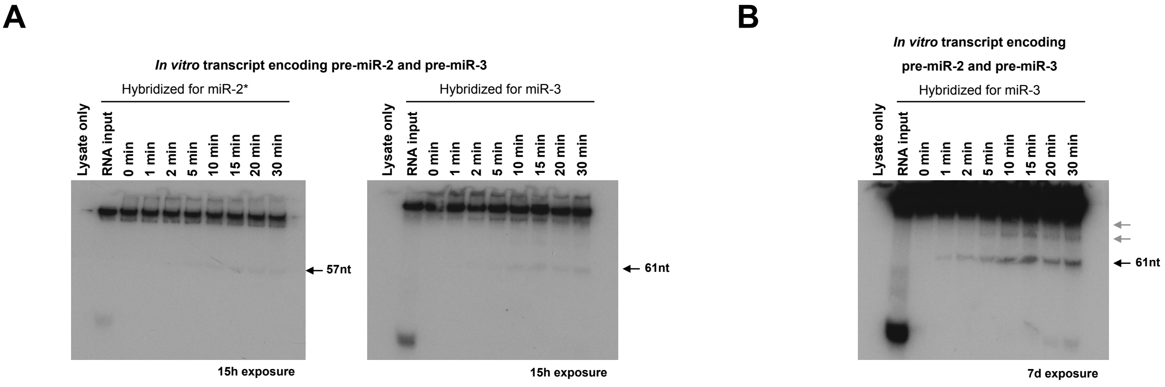

Figure S2. *In vitro* Microprocessor cleavage assays of pre-miR-BHRF1-2 and -3.

(A) We performed an *in vitro* Microprocessor cleavage assay with a transcript encoding pre-miR-BHRF1-2 and -3. The assay was stopped at the indicated length of time. The samples were separated on a PAGE, blotted, and hybridized with probes specific for miR-BHRF1-2* and for miR-BHRF1-3. A non-treated RNA input and a lysate only control were also loaded. A synthetic miRNA (50 fmol) was loaded in the RNA input lane to normalize for differences in hybridization efficiency and allow comparison between the two Northern blots. **(B)** We also show a longer exposure of the Northern blot hybridized with a probe specific for miR-BHRF1-3 cleavage that reveals intermediate cleavage products (grey arrows), as well as a smear that encompasses large forms of the transcript.

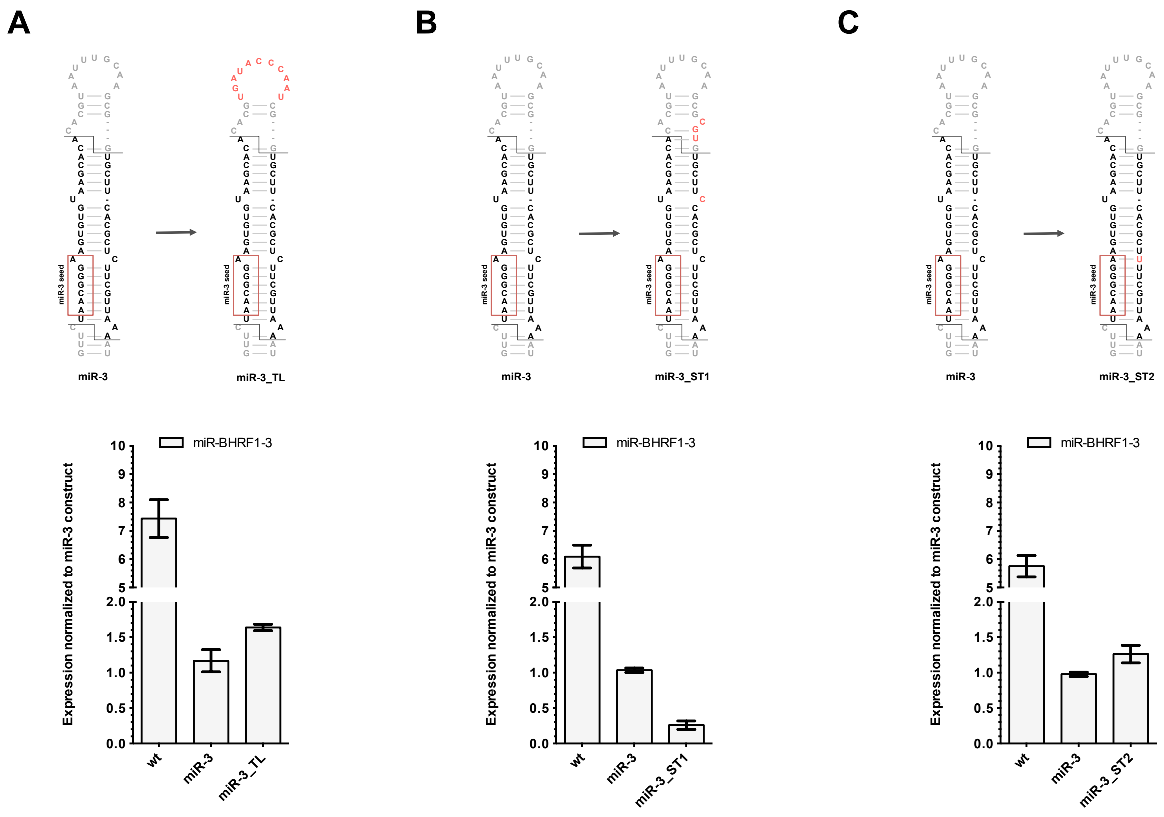

Figure S3. Mutation analysis of miR-BHRF1-3 sequence elements.

To delineate the influence of various pre-miR-BHRF1-3 domains on the efficiency of processing, these were mutated and miR-BHRF1-3 expressed in HEK293 cells. The expression levels of these mutants relative to intact miR-BHRF1-3 and to the complete BHRF1 miRNA locus (wt, described in Figure 1A) are shown. The mutants include a construct in which the terminal loop of miR-BHRF1-3 was exchanged with 11 nucleotides of the terminal loop of miR-BHRF1-2 **(A)**, a construct in which single-stranded nucleotides were complemented in order to achieve a straight stem-loop structure **(B)**, and a construct in which an unpaired A-C bulge at the lower extremity of the stem was corrected **(C)**. (TL = terminal loop, ST = stem-loop). Results show average values from triplicate transfections ± s.d.

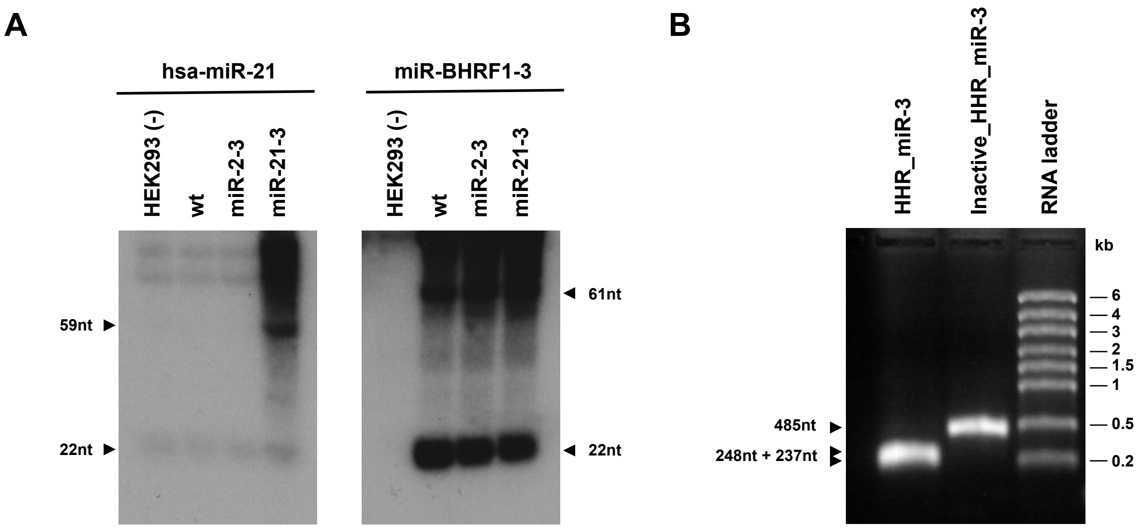

Figure S4. Confirmation of hsa-miR-21 expression and HHR functionality.

(A) MiRNA Northern blots with probes specific for hsa-miR-21 and miR-BHRF1-3. Negative controls included mock-transfected HEK293 cells. **(B)** We confirmed the autocatalytic activity of *in vitro* transcribed Hammerhead ribozyme (HHR) RNA by loading the HHR and its inactive variant on a 1.5% agarose gel. The gel was stained with ethidium bromide after electrophoresis.

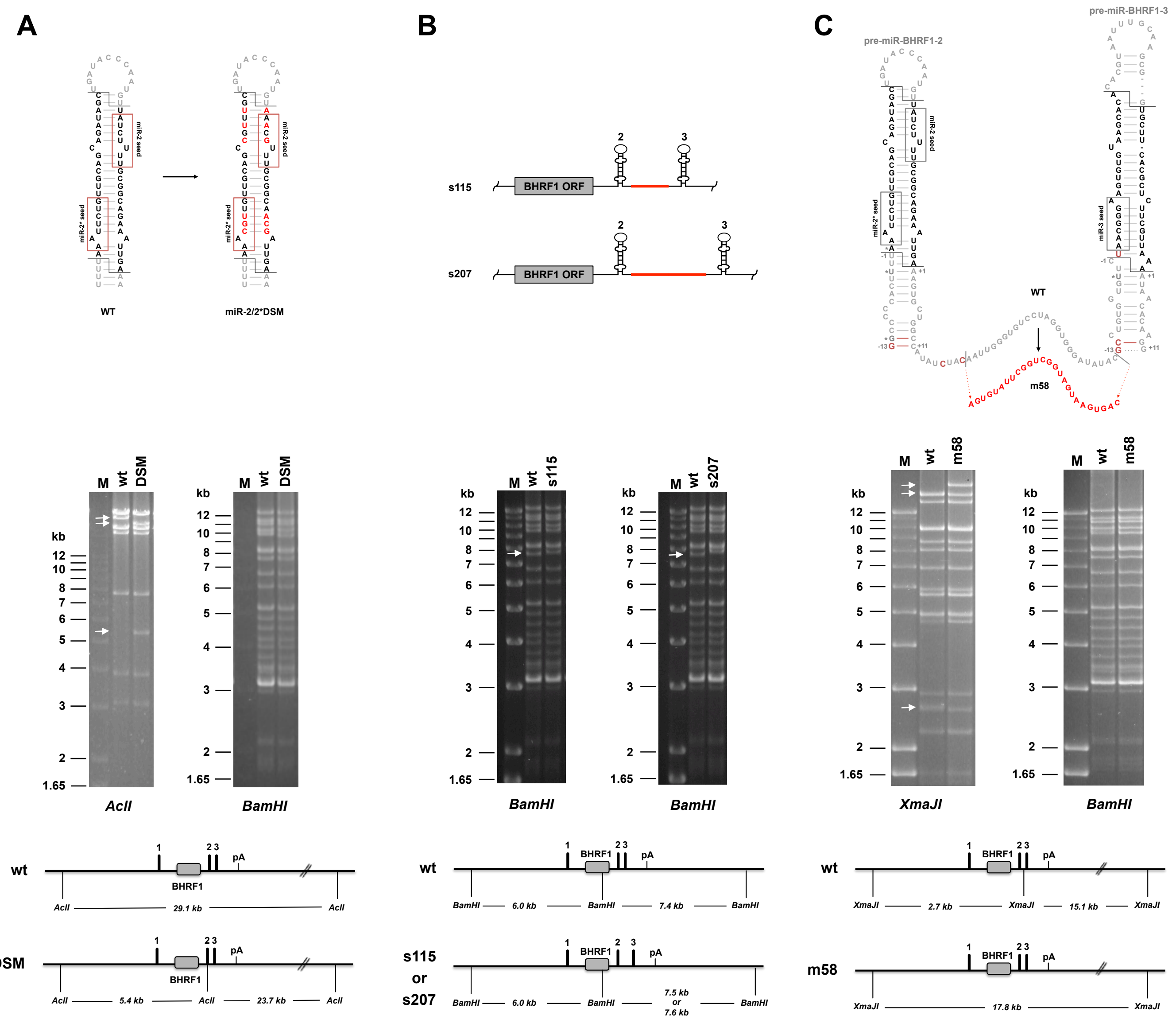

Figure S5. Generation of recombinant EBV BACs.

(A) Double seed mutant (DSM) bacterial artificial chromosome (BAC) construction. Schematic overview of the mutations introduced within the seed regions of pre-miR-BHRF1-2/2*, which are highlighted in red. Complementary nucleotides on opposite strands were mutated in order to maintain the overall stem-loop integrity. Introduced mutations were designed to yield a new AclI-cleavage site within the BHRF1 locus. Cleavage of the mutant with AclI therefore results in two bands of 23.7 kb and 5.4 kb after agarose gel electrophoresis, while only a single 29.1 kb band is generated from the wt genome (shifted bands are highlighted with white arrows). The genome integrity of the cloned DSM BAC was confirmed by BamHI digestion, where the band pattern remains unchanged outside the introduced mutations. **(B)** Schematic overview of EBV BACs carrying the 115nt or 207nt spacer sequence (red) inserted between miR-BHRF1-2 and -3. A BamHI digestion of the mutant BACs reveals a fragment shifted from 7.4 kb to 7.5 kb or 7.6 kb on an agarose gel (highlighted with a white arrow), which corresponds to the addition of 115nt or 207nt, respectively. **(C)** The sequence mutated in the genome of the m58 EBV mutant is highlighted in red. This mutation deleted an XmaJI cleavage site within a 17.8 kb fragment that exists as a 2.7 kb and a 15.1 kb fragment in the wt BAC genome. The genome integrity of this mutant was confirmed by BamHI digestion.

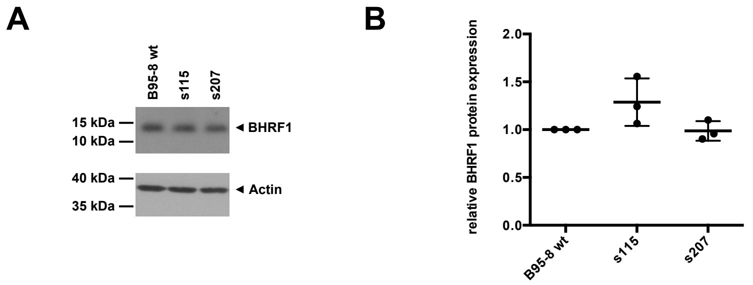

Figure S6. BHRF1 protein levels in LCLs infected with s115 and s207 EBV.

We assessed expression of the BHRF1 protein by Western blot in three independent sets of LCLs infected with either of the mutants carrying an artificial spacer in the BHRF1 microRNA locus. LCLs generated with the B95-8 wild type virus served as a reference. We used a mouse monoclonal antibody to detect BHRF1 and an antibody against actin as a loading control. Signals were scanned and quantified using the ImageJ software. **(A)** shows a representative Western blot. **(B)** This dotplot shows quantification of BHRF1 expression in three sets of LCLs (26-28dpi).

SUPPLEMENTARY EXPERIMENTAL PROCEDURES

Construction of microRNA expression plasmids

All expression plasmids used in this study were cloned by inserting the BHRF1 sequence fragments into the multiple cloning site of pcDNA3.1 (+). A description of plasmid insert sequences and cloning details is provided in Supplementary Table S1. The identity of the inserted sequences was confirmed by sequencing (MWG Eurofins). Plasmid DNA for transfection experiments was isolated with the Jetstar 2.0 Midiprep kit (Genomed).

miRNA quantification by stem-loop RT-PCR

To determine absolute miRNA values, standard curves for stem-loop RT-PCR were generated for all BHRF1 miRNAs with defined amounts of synthetic miRNAs. Sequences of miRNA oligonucleotides (purchased from MWG Eurofins) are depicted in Supplementary Table S2. Expression levels were calculated per pg total RNA based on Ct_{mean} values.

miRNA quantification by Northern blot

Quantification of BHRF1 miRNAs by Northern blot was performed to compare expression levels in LCLs with transfected HEK293 cells. Defined amounts of synthetic miRNAs and 15µg cellular RNA were loaded on a 15% Mini-Protean TBE-urea acrylamide gels (BioRad) and run at 80V for 120min. After transfer on a Hybond-N+ membrane (GE Healthcare) by semi-dry blotting in TBE at 25V for 2.5h, RNAs were UV-crosslinked with the membrane (1200 µJoules) and hybridized with [^{32}P]-labeled oligonucleotide probes in ExpressHyb solution (ClonTech) at 37°C for 1h. After washing twice in 2x SSC, 0.05% SDS for 15min and in 0.1x SSC, 0.1% SDS for 15min, blots were dried and signals detected on Hyperfilm MP (GE Healthcare) after exposure at -80°C for one to ten days to achieve similar signal intensities for all blots.

All other miRNA Northern blots shown in this publication were performed according to the same experimental procedures. A full list of used synthetic miRNAs and Northern blot probes is provided in Supplementary Table S2.

Construction of EBV mutants

All virus coordinates are related to the sequence of B95-8 EBV strain, accession number V01555.2. The genome of B95-8 EBV has previously been cloned as a bacterial artificial chromosome (BAC), which contains chloramphenicol and hygromycin resistance genes as well as a gene for green fluorescent protein (GFP) expression (1).

Mutations within the stem-loop of miR-BHRF1-2 to obtain the double seed mutant (miR-2/2*DSM) were introduced into the B95-8 BAC by chromosomal building as previously described (2,3). A targeting vector for chromosomal building was cloned by insertion of the BHRF1 gene (#53720-55389) with correspondingly modified seed regions into the XhoI site of shuttle vector B269.

Successfully recombined clones were screened by AclI cleavage and genome integrity was monitored by BamHI digestion.

The 115 nucleotides spacer sequence (5' C - CTAGGCTGCA GATCCAGTCT ACAGTGTTAT CACTCAGTCA TGCCATATCT CCTGTCATCT CACCTTGCTC CTGCCGAGAA AGTATCCATC ATGGCTGATG CAATGCTTGG GTGTC - CTAGG 3') was introduced between miR-BHRF1-2 and -3 via an XmaJI cleavage site (#55227-55232). The resulting plasmid served as a template for *En passant* mutagenesis (4) of an EBV. Briefly, a kanamycin resistance cassette was introduced into the spacer region and the resulting fragment inserted by electroporation into *E. coli* GS1783-B95-8 cells. Successfully recombined clones were selected on agar plates with 50µg/ml kanamycin. The resistance gene was removed in a second round of homologous recombination to yield the 115nt_spacer EBV mutant, termed EBV s115, whose structural integrity was confirmed by BamHI digestion.

A similar strategy was followed to construct EBV s207 that contains a 207nt spacer sequence (5' C - CTAGGGGATT GCACGCATGT TCTCCGGCCG CTTGGGTGGA GAGGCTATTC GGCTATGACT GGGCACAACA GACAATCGGC TGCTCTGATG CCGCCGTGTT CCGGCTGTCA GCGCAGGGGC GCCCGGTTCT TTTTGTCAAG ACCGACCTGT CCGGTGCCCT GAATGAACTG CAGGACGAGG CAGCGCGGCT ATCGTGGCTG GCCACGC - CTAGG 3'). We followed a similar strategy to introduce a mutated version of the 58nt sequence into a recombinant virus. In this case, we shuffled 24 of the nucleotides at position 55217-55242 (5' - AATTGGGTGT CCTAGGTGGG ATATAC - 3'). The resulting mutant was termed EBV m58.

Generation of viral supernatants and viral titer determination

Stable cell lines for production of virus supernatants were established by transfecting $3x10^5$ HEK293 cells with 1.5µg of mutant BAC DNA using Metafectene (Biontex). One day after transfection, cells were seeded on 150mm cell culture dishes in RPMI 1640 medium with 10% FBS and 100µg/ml hygromycin for selection of clones containing an EBV genome. GFP-positive colonies were picked 3 weeks later, tested for production of progeny virus upon induction and used for subsequent experiments. To trigger release of virions into cell culture supernatant, producer cells were seeded at a density of $3x10^5$ cells per 6-well and induced by transient transfection of 0.5µg BZLF1 and BALF4 expression plasmids using Metafectene. Medium was replaced with fresh RPMI 10% FBS the next day, supernatants harvested at day 4 post-induction and filtered through a 0.45µm filter. Concentration of viral particles in supernatants was assessed by TaqMan Real-Time PCR quantification of viral DNA polymerase BALF5 with primer sequences and sample treatment as described previously (5). The Step One Plus™ Real-Time PCR system (Applied Biosystems) was used for measurements. Viral titers per ml supernatant were calculated based on a standard curve from a serial dilution of BAC DNA with defined concentration.

To determine titers of infectious virions, 10^5 Raji cells were infected in 96-U wells with increasing 5-fold dilutions of viral supernatants. The percentage of GFP-positive cells was counted three days post-infection and titers calculated in green Raji units (gru) per ml.

BrdU incorporation assay

Cell cycle analysis of proliferating B cells after infection with wt or mutant EBV was performed with the APC BrdU flow kit (BD Pharmingen). Briefly, 10^6 cells were exposed to 10mM BrdU for 30min at 37°C, 5% CO_2. Incorporation was detected after DNAse treatment of cells by immunostaining with an APC-coupled antibody against BrdU. Additionally, DNA content was determined by staining with 7-AAD. Cells were analyzed on a FACSCalibur™ flow cytometer and subpopulations distinguished according to G_0/G_1 (BrdU negative, single DNA content), S (BrdU positive) and G_2/M phase (BrdU negative, double DNA content).

Western blot

Lymphoblastoid cell lines were harvested by centrifugation, pellets washed once in PBS and lysed in RIPA buffer after addition of a protease inhibitor cocktail (1:1000, Sigma). Lysates were sonicated and 50µg protein separated on 15% SDS-polyacrylamide gels, then transferred on a nitrocellulose membrane (0.45µm NC, Amersham) by wet blotting. Membranes were incubated with antibodies against BHRF1 (mouse monoclonal 3E8, 1:100, kindly provided by J.-Y. Chen (6)) and Actin (mouse monoclonal ACTN05, 1:10.000, Dianova) followed by a secondary antibody conjugated to horseradish peroxidase (goat anti-mouse-IgG, w402b, 1:40.000, Promega) and ECL detection (Perkin Elmer). Immunoblots were quantified using ImageJ 1.45s software.

Supplementary Table S1. Cloning details of microRNA expression plasmids.

Plasmid name	Position of BHRF1 fragment within B95-8 genome (V01555.2)	Cloning description	Comments
wt	53720-55389	Cloned into XhoI site of pcDNA3.1 (+)	Encompasses entire BHRF1 locus
miR-2	55049-55255	Cloned into EcoRI site of pcDNA3.1 (+)	miR-BHRF1-2 expression plasmid
miR-3	55198-55395	Cloned into EcoRI site of pcDNA3.1 (+)	miR-BHRF1-3 expression plasmid
miR-2-3	55049-55395	Cloned into EcoRI site of pcDNA3.1 (+)	miR-BHRF1-2 and -3 expression plasmid
miR-21-3	Same as miR-2-3, nucleotides 55135-55201 corresponding to pre-miR-BHRF1-2 replaced by pre-hsa-miR-21 sequence	Ordered as synthetic DNA sequence from MWG Eurofins and cloned into EcoRI site of pcDNA3.1 (+)	Introduced pre-hsa-miR-21 sequence:<br>5'-TCGGGTAGCT TATCAGACTG ATGTTGACTG TTGAATCTCA TGGCAACACC AGTCGATGGG CTGTCTGA-3'
HHR_miR-3	Same as miR-2-3, nucleotides 55134-55202 corresponding to miR-BHRF1-2 stem-loop replaced by a hammerhead ribozyme (HHR) sequence	Sequence exchanged by PCR-based site-directed mutagenesis with miR-2-3 plasmid as template	Introduced HHR sequence:<br>5'- CTGAGGTGCA GGTACATCCA GCTGACGAGT CCCAAATAGG ACGAAACGCG CTTCGGTGCG TCCTGGATTC CACTGCTATC CAC-3'<br>PCR-primer:<br>HHR_fwd: 5'-CGAAACGCGC TTCGGTGCGT CCTGGATTCC ACTGCTATCC ACTGGCCATA TCTACAATTG GGTGTCC-3'<br>HHR_rev: 5'- TCCTATTTGG GACTCGTCAG CTGGATGTAC CTGCACCTCA GGGGCCGGGT CATTGGCATG TTATTCTTGT AAGCC-3'
Inactive-HHR_miR-3	Same as for active ribozyme	Sequence exchanged by PCR-based site-directed mutagenesis with miR-2-3 plasmid as template; ribozyme cleavage-deficient due to a point mutation within cleavage site (highlighted in red).	Introduced HHR sequence:<br>5'- CTGAGGTGCA GGTACATCCA GCTGACGAGT CCCAAATAGG ACGAGACGCG CTTCGGTGCG TCCTGGATTC CACTGCTATC CAC-3'<br>PCR-primer:<br>HHR_fwd_inact: 5'- CGAGACGCGC TTCGGTGCGT CCTGGATTCC ACTGCTATCC ACTGGCCATA TCTACAATTG GGTGTCC-3'<br>HHR_rev: 5'- TCCTATTTGG GACTCGTCAG CTGGATGTAC CTGCACCTCA GGGGCCGGGT CATTGGCATG TTATTCTTGT AAGCC-3'
miR-3_ TL	Same as miR-3, nucleotides 55282-55292 corresponding to terminal loop of miR-3 replaced by nucleotides 55163-55173 corresponding to terminal loop of miR-2	Ordered as synthetic DNA sequence from MWG Eurofins and cloned into EcoRI site of pcDNA3.1 (+)	-
miR-3 _ST1	Same as miR-3, additional nucleotides inserted in miR-BHRF1-3 stem-loop as shown in Figure S3	Ordered as synthetic DNA sequence from MWG Eurofins and cloned into EcoRI site of pcDNA3.1 (+)	-
miR-3 _ST2	Same as miR-3, point mutation at position 55307 (C-T) as shown in Figure S3	Ordered as synthetic DNA sequence from MWG Eurofins and cloned into EcoRI site of pcDNA3.1 (+)	-

miR-2 ΔpA<br>miR-3 ΔpA<br>miR-2-3 ΔpA	Same as miR-2 / miR-3 / miR-2-3	“AAU AAA” RNA cleavage signal within BGH polyA site of corresponding plasmids deleted by PCR-based site-directed mutagenesis	PCR-primer:<br>dpA_fwd: 5’-ATGAGGAAAT TGCATCGCAT TGTCTGAGTA GG-3’<br>dpA_rev: 5’-AGGAAAGGAC AGTGGGAGTG GCACCTTCCA GG-3’
miR-2_ β-lac	Nucleotides 55117-55219 corresponding to miR-BHRF1-2 stem-loop flanked on each side by 100 nt of β-lactamase gene sequences	Ordered as synthetic DNA sequence from MWG Eurofins and cloned into EcoRI site of pcDNA3.1 (+)	5’ flanking sequence:<br>5’-ATACCGCGAG ACCCACGCTC ACCGGCTCCA GATTTATCAG CAATAAACCA GCCAGCCGGA AGGGCCGAGC GCAGAAGTGG TCCTGCAACT TTATCCGCCT-3’<br>3’ flanking sequence:<br>5’-CCATCCAGTC TATTAATTGT TGCCGGGAAG CTAGAGTAAG TAGTTCGCCA GTTAATAGTT TGCGCAACGT TGTTGCCATT GCTACAGGCA TCGTGGTGTC-3’
miR-3_ β-lac	Nucleotides 55231-55339 corresponding to miR-BHRF1-3 stem-loop flanked on each side by 100 nt of β-lactamase gene sequences	Ordered as synthetic DNA sequence from MWG Eurofins and cloned into EcoRI site of pcDNA3.1 (+)	Same as for miR-2_ β-lac
pre-miR-2_base_miR-3	Nucleotides 55141-55197 corresponding to pre-miR-BHRF1-2 flanked by nucleotides 55198-55255 and 55317-55395 of pre-miR-BHRF1-3 surrounding sequence elements	Ordered as synthetic DNA sequence from MWG Eurofins and cloned into EcoRI site of pcDNA3.1 (+)	-
pre-miR-3_base_miR-2	Nucleotides 55256-55316 corresponding to pre-miR-BHRF1-3 flanked by nucleotides 55049-55140 and 55198-55255 of pre-miR-BHRF1-2 surrounding sequence elements	Ordered as synthetic DNA sequence from MWG Eurofins and cloned into EcoRI site of pcDNA3.1 (+)	-
miR-2-3_exchange	Based on miR-2-3, nucleotides 55092-55208 encompassing miR-BHRF1-2 stem-loop exchanged with nucleotides 55211-55364 containing miR-BHRF1-3 stem-loop	Ordered as synthetic DNA sequence from MWG Eurofins and cloned into EcoRI site of pcDNA3.1 (+)	-
miR-3_115nt	Same as miR-3, nucleotides 55227-55232 (XmaJI cleavage site) used for insertion of spacer sequence	Spacer fwd and rev fragments annealed and ligated via XmaJI cleavage site of miR-3 expression plasmid	Inserted spacer fragments:<br>115nt_fwd: 5’-CTAGGCTGCA GATCCAGTCT ACAGTGTTAT CACTCAGTCA TGCCATATCT CCTGTCATCT CACCTTGCTC CTGCCGAGAA AGTATCCATC ATGGCTGATG CAATGCTTGG GTGTC-3’<br>115nt_rev: 5’-CTAGGACACC CAAGCATTGC ATCAGCCATG ATGGATACTT TCTCGGCAGG AGCAAGGTGA GATGACAGGA GATATGGCAT GACTGAGTGA TAACACTGTA GACTGGATCT GCAGC-3’
miR-2-3_115nt	Same as miR-2-3, nucleotides 55227-55232 (XmaJI cleavage site) used for insertion of spacer sequence	Spacer fwd and rev fragments annealed and ligated via XmaJI cleavage site of miR-2-3 expression plasmid	Same as for miR-3_115nt
miR-2-3_115nt_inverse	Same as for miR-2-3_115nt	Spacer insert of miR-2-3_115nt ligated in inverse orientation	Same as for miR-2-3_115nt, ligated in inverse orientation

miR-2-3_207nt	Same as miR-2-3, nucleotides 55227-55232 (XmaJI cleavage site) used for insertion of spacer sequence	Spacer sequence amplified by PCR from a Neomycin resistance cassette and ligated via XmaJI cleavage site of miR-2-3 expression plasmid	Inserted spacer sequence:<br>5'-CTAGGGGATT GCACGCATGT TCTCCGGCCG CTTGGGTGGA GAGGCTATTC GGCTATGACT GGGCACAACA GACAATCGGC TGCTCTGATG CCGCCGTGTT CCGGCTGTCA GCGCAGGGGC GCCCGGTTCT TTTTGTCAAG ACCGACCTGT CCGGTGCCCT GAATGAACTG CAGGACGAGG CAGCGCGGCT ATCGTGGCTG GCCACGC-3'
miR-2-3_775nt	Same as miR-2-3, nucleotides 55227-55232 (XmaJI cleavage site) used for insertion of spacer sequence	Spacer sequence amplified by PCR from a Neomycin resistance cassette and ligated via XmaJI cleavage site of miR-2-3 expression plasmid	Inserted spacer sequence:<br>5'-CTAGGGGATT GCACGCAGGT TCTCCGGCCG CTTGGGTGGA GAGGCTATTC GGCTATGACT GGGCACAACA GACAATCGGC TGCTCTGATG CCGCCGTGTT CCGGCTGTCA GCGCAGGGGC GCCCGGTTCT TTTTGTCAAG ACCGACCTGT CCGGTGCCCT GAATGAACTG CAGGACGAGG CAGCGCGGCT ATCGTGGCTG GCCACGACGG GCGTTCCTTG CGCAGCTGTG CTCGACGTTG TCACTGAAGC GGGAAGGGAC TGGCTGCTAT TGGGCGAAGT GCCGGGGCAG GATCTCCTGT CATCTCACCT TGCTCCTGCC GAGAAAGTAT CCATCATGGC TGATGCAATG CGGCGGCTGC ATACGCTTGA TCCGGCTACC TGCCCATTCG ACCACCAAGC GAAACATCGC ATCGAGCGAG CACGTACTCG GATGGAAGCC GGTCTTGTCG ATCAGGATGA TCTGGACGAA GAGCATCAGG GGCTCGCGCC AGCCGAACTG TTCGCCAGGC TCAAGGCGCG CATGCCCGAC GGCGAGGATC TCGTCGTGAC CCATGGCGAT GCCTGCTTGC CGAATATCAT GGTGGAAAAT GGCCGCTTTT CTGGATTCAT CGACTGTGGC CGGCTGGGTG TGGCGGACCG CTATCAGGAC ATAGCGTTGG CTACCCGTGA TATTGCTGAA GAGCTTGGCG GCGAATGGGC TGACCGCTTC CTCGTGCTTT ACGGTATCGC CGCTCCCGAT TCGCAGCGCA TCGCCTTCTA TCGCCTTCTT GACGC-3'
miR-3_m58	Same as miR-3, nucleotides 55217-55242 replaced by a shuffled version of the sequence	Ordered as synthetic DNA sequence from MWG Eurofins and cloned into EcoRI site of pcDNA3.1 (+)	Shuffled sequence:<br>5' - AATTGGGTGT CCTAGGTGGG ATATAC - 3'
miR-2-3_m58	Same as miR-2-3, nucleotides 55217-55242 replaced by a shuffled version of the sequence	Ordered as synthetic DNA sequence from MWG Eurofins and cloned into EcoRI site of pcDNA3.1 (+)	Shuffled sequence:<br>5' - AATTGGGTGT CCTAGGTGGG ATATAC - 3'

Supplementary Table S2. Oligonucleotides for microRNA quantification and Northern blot probes.

**microRNA**	**Length**	**5' – sequence – 3'**
miR-BHRF1-1	22nt	UAACCUGAUCAGCCCCGGAGUU
miR-BHRF1-2	22nt	UAUCUUUUGCGGCAGAAAUUGA
miR-BHRF1-2*	22nt	AAAUUCUGUUGCAGCAGAUAGC
miR-BHRF1-3	22nt	UAACGGGAAGUGUGUAAGCACA
**microRNA probe**	**Length**	**5' – sequence – 3'**
miR-1.comp	22nt	AACTCCGGGGCTGATCAGGTTA
miR-2.comp	22nt	TCAATTTCTGCCGCAAAAGATA
miR-2*.comp	22nt	GCTATCTGCTGCAACAGAATTT
miR-3.comp	22nt	TGTGCTTACACACTTCCCGTTA
miR-21.comp	22nt	TCAACATCAGTCTGATAAGCTA
58_nt.comp	20nt	TGTAGATATGGCCAGCACTT

Supplementary Table S3. ***In vitro*** **RNA (IVR) transcript sequences.**

Name	Length	Transcript sequence
IVR miR-2	346nt	TAATACGACTCACTATA --- GGGAGACCCA AGCTGGCTAG CGTTTAAACT TAAGCTTGGT ACCGAGCTCG GATCCACTAG TCCAGTGTGG TGGAATTCAC TAGTGATTTT CTGGCCTCAC TGGCCGCATT ATAATTTAAC CAAACAGTGG TCGTGAGTTT TAGGCCGGCC ATGGGGGCTT ACAAGAATAA CATGCCAATG ACCCGGCCCC CACTTTTAAA TTCTGTTGCA GCAGATAGCT GATACCCAAT GTTATCTTTT GCGGCAGAAA TTGAAAGTGC TGGCCATATC TACAATTGGG TGTCCTAGGT GGGATATACG CCTGTGGTGT TCGGCCAGTG AGGCCTATAA ATCGAATTCT GCAGAT
IVR miR-3	335nt	TAATACGACTCACTATA --- GGGAGACCCA AGCTGGCTAG CGTTTAAACT TAAGCTTGGT ACCGAGCTCG GATCCACTAG TCCAGTGTGG TGGAATTCGA TTTCTGGCCT CACTGGCCAA GTGCTGGCCA TATCTACAAT TGGGTGTCCT AGGTGGGATA TACGCCTGTG GTGTTCTAAC GGGAAGTGTG TAAGCACACA CGTAATTTGC AAGCGGTGCT TCACGCTCTT CGTTAAAATA ACACAAGGAC AAGATACTAA AGAAATAACT GAGGTGAGTG TGGGAAGATG GGAATACTAT GTGTTATGTT AACGGGGGCC AGTGAGGCCT ATAATCACTA GTGAATTCTG CAGAT
IVR miR-2-3	485nt	TAATACGACTCACTATA --- GGGAGACCCA AGCTGGCTAG CGTTTAAACT TAAGCTTGGT ACCGAGCTCG GATCCACTAG TCCAGTGTGG TGGAATTCGA TTTATAGGCC TCACTGGCCG CATTATAATT TAACCAAACA GTGGTCGTGA GTTTTAGGCC GGCCATGGGG GCTTACAAGA ATAACATGCC AATGACCCGG CCCCCACTTT TAAATTCTGT TGCAGCAGAT AGCTGATACC CAATGTTATC TTTTGCGGCA GAAATTGAAA GTGCTGGCCA TATCTACAAT TGGGTGTCCT AGGTGGGATA TACGCCTGTG GTGTTCTAAC GGGAAGTGTG TAAGCACACA CGTAATTTGC AAGCGGTGCT TCACGCTCTT CGTTAAAATA ACACAAGGAC AAGATACTAA AGAAATAACT GAGGTGAGTG TGGGAAGATG GGAATACTAT GTGTTATGTT AACGGGGGCC AGTGAGGCCT ATAATCACTA GTGAATTCTG CAGAT

Precursor sequences of corresponding microRNAs are highlighted in red. The binding site for SHAPE primers is identical for all three transcripts and is shown in orange. The T7 promoter sequence is not part of RNA transcripts, but is added in grey letters to complete the description. IVR = *in vitro* RNA.

SUPPLEMENTARY REFERENCES

1. Delecluse, H.J., Hilsendegen, T., Pich, D., Zeidler, R. and Hammerschmidt, W. (1998) Propagation and recovery of intact, infectious Epstein-Barr virus from prokaryotic to human cells. *Proceedings of the National Academy of Sciences of the United States of America*, **95**, 8245-8250.

2. Neuhierl, B. and Delecluse, H.J. (2005) Molecular genetics of DNA viruses: recombinant virus technology. *Methods in molecular biology*, **292**, 353-370.

3. Feederle, R., Bartlett, E.J. and Delecluse, H.J. (2010) Epstein-Barr virus genetics: talking about the BAC generation. *Herpesviridae*, **1**, 6.

4. Tischer, B.K., Smith, G.A. and Osterrieder, N. (2010) En passant mutagenesis: a two step markerless red recombination system. *Methods in molecular biology*, **634**, 421-430.

5. Feederle, R., Bannert, H., Lips, H., Muller-Lantzsch, N. and Delecluse, H.J. (2009) The Epstein-Barr virus alkaline exonuclease BGLF5 serves pleiotropic functions in virus replication. *Journal of virology*, **83**, 4952-4962.

6. Chou, S.P., Tsai, C.H., Li, L.Y., Liu, M.Y. and Chen, J.Y. (2004) Characterization of monoclonal antibody to the Epstein-Barr virus BHRF1 protein, a homologue of Bcl-2. *Hybridoma and hybridomics*, **23**, 29-37.
